# Fenton/Fenton-like metal-based nanomaterials combine with oxidase for synergistic tumor therapy

**DOI:** 10.1186/s12951-021-01074-1

**Published:** 2021-10-16

**Authors:** Wei Cao, Mengyao Jin, Kang Yang, Bo Chen, Maoming Xiong, Xiang Li, Guodong Cao

**Affiliations:** 1 Department of General Surgery, First Affiliated Hospital of Anhui Medical University, Hefei, 230022 People’s Republic of China; 2 Department of General Surgery, The Fourth Affiliated Hospital of Anhui Medical University, Hefei, 230022 People’s Republic of China; 3 State Key Laboratory of Silicon Materials, School of Materials Science and Engineering, Zhejiang University, Hangzhou, 310027 People’s Republic of China

**Keywords:** Chemodynamic therapy, Metabolite oxidase, Tumor synergistic therapy, Fenton reaction, Immunotherapy

## Abstract

Chemodynamic therapy (CDT) catalyzed by transition metal and starvation therapy catalyzed by intracellular metabolite oxidases are both classic tumor treatments based on nanocatalysts. CDT monotherapy has limitations including low catalytic efficiency of metal ions and insufficient endogenous hydrogen peroxide (H_2_O_2_). Also, single starvation therapy shows limited ability on resisting tumors. The “metal-oxidase” cascade catalytic system is to introduce intracellular metabolite oxidases into the metal-based nanoplatform, which perfectly solves the shortcomings of the above-mentioned monotherapiesIn this system, oxidases can not only consume tumor nutrients to produce a “starvation effect”, but also provide CDT with sufficient H_2_O_2_ and a suitable acidic environment, which further promote synergy between CDT and starvation therapy, leading to enhanced antitumor effects. More importantly, the “metal-oxidase” system can be combined with other antitumor therapies (such as photothermal therapy, hypoxia-activated drug therapy, chemotherapy, and immunotherapy) to maximize their antitumor effects. In addition, both metal-based nanoparticles and oxidases can activate tumor immunity through multiple pathways, so the combination of the “metal-oxidase” system with immunotherapy has a powerful synergistic effect. This article firstly introduced the metals which induce CDT and the oxidases which induce starvation therapy and then described the “metal-oxidase” cascade catalytic system in detail. Moreover, we highlight the application of the “metal-oxidase” system in combination with numerous antitumor therapies, especially in combination with immunotherapy, expecting to provide new ideas for tumor treatment.

## Introduction

In the twenty-first century, tumors are considered as one of the main threats to human health [1]. With the increasing aging of the Chinese population, tumors have grown up to be a major public health problem threatening Chinese residents [2, 3]. Traditional tumor treatment methods mainly include surgery, chemotherapy, and radiotherapy. However, chemotherapy and radiotherapy are not targeted and always cause accompanied damage to normal cells or organs. Therefore, there is an urgent need for a treatment strategy with stable efficacy and high biological safety. This strategy is expected to have the following characteristics: (1) It can effectively kill the tumor in situ. (2) It can inhibit tumor metastasis. (3) It can achieve tumor-targeted therapy. (4) It has lower toxicity and less adverse effects. Nanomedicine is the application of the principles and methods of nanoscience into medicine. In recent years, increasing nanomaterials have been used for tumor treatment, and various novel tumor treatments derived from nanomedicine have brought hopes for overcoming malignant tumors [4, 5].

There are complex and diverse mechanisms of using nanomaterials to kill tumor cells. An important theoretical basis is that nanoparticles (NPs) loading functional factors react within tumor cells and further generate a large amount of strong oxidative reactive oxygen species (ROS), which result in tumor cell death. In human body, most of the strong oxidizing hydroxy radical (•OH) derive from the catalytic decomposition of H_2_O_2_ [6] induced by metal ions, and the most famous metal ion is Fe^2+^. The main reaction process can be concluded as: under acidic conditions, the chain reaction between Fe^2+^ and H_2_O_2_ catalyzes the formation of •OH, which is the so-called Fenton reaction. Besides Fe^2+^, other transition metal ions (such as Cu^2+^ and Mn^2+^) can also catalyze similar chemical reactions, referred to Fenton-like reaction. The antitumor therapy based on Fenton/Fenton-like reaction is called CDT. The bases of applying CDT in tumor treatment are summarized as follows: (1) Due to the enhanced permeability and retention (EPR) effect, nanoparticles mainly accumulate in the tumor microenvironment (TME), but rarely in normal tissues. (2) H_2_O_2_ excessively accumulate in the TME (100 uM to 1 mM), while the H_2_O_2_ concentration in normal tissues is extremely low. (3) Excessive ROS can be produced in the acidic TME due to the facilitation of the Fenton reaction, while few ROS can be produced in a neutral environment.

On the other hand, tumors have a special metabolic pattern, such as the large demand of glucose for anaerobic glycolysis, which leads to the accumulation of lactate in the TME. Glucose oxidase (GOx), lactate oxidase (LOX) and amino acid oxidase (AAO) can oxidize and decompose correlated nutrients, further increasing the level of strong oxidizing H_2_O_2_ [7–10]. The reduction of glucose, lactate, and amino acid levels can effectively block the energy supply of tumor cells to "starve" tumor cells, also regarded as tumor starvation therapy [11]. At the same time, the excessive H_2_O_2_ produced in the tumor can be further converted into more toxic •OH through the Fenton/Fenton-like reaction or under laser irradiation, which will eventually lead to the valid elimination of tumor cells. Therefore, “metal-oxidase” cascade catalytic system formed by metal-based nanoparticles loaded with oxidases can ensure not only the antitumor effect but also the biosafety, considering as a promising tumor treatment strategy. In addition, the “metal-oxidase” system can combine with photothermal therapy (PTT), hypoxia activated therapy, chemotherapy and immunotherapy to perform a more powerful antitumor effect.

Tumor metastasis and recurrence are the major causes of tumor lethality, which can’t be solved by existing antitumor therapies such as CDT, PTT, starvation therapy and so on. Recent evidence suggests that tumor immunotherapy can not only eliminate tumors in situ but also effectively inhibit metastasis and recurrence [12, 13]. However, the low immune response of the tumor limits the applications of immunotherapy [14, 15]. Both metal-based nanoparticles and oxidases can further promote the maturation of dendritic cells (DCs) and activate tumor immunity by inducing tumor cell death and releasing tumor-associated antigens (TAAs) [16–18]. The “metal-oxidase” system can thoroughly integrate the advantages of them. On the one hand, it can induce tumor cell death and promote immune initiation through various ways such as ferroptosis and apoptosis. On the other hand, it can accelerate the immune process by reversing the immunosuppressive TME (such as reducing lactic acid accumulation and acidity) [19, 20]. Therefore, the combination of “metal-oxidase” system and immunotherapy has a strong synergistic effect, which can not only eliminate the tumor in situ, but also inhibit the recurrence and metastasis.

**Scheme 1:**
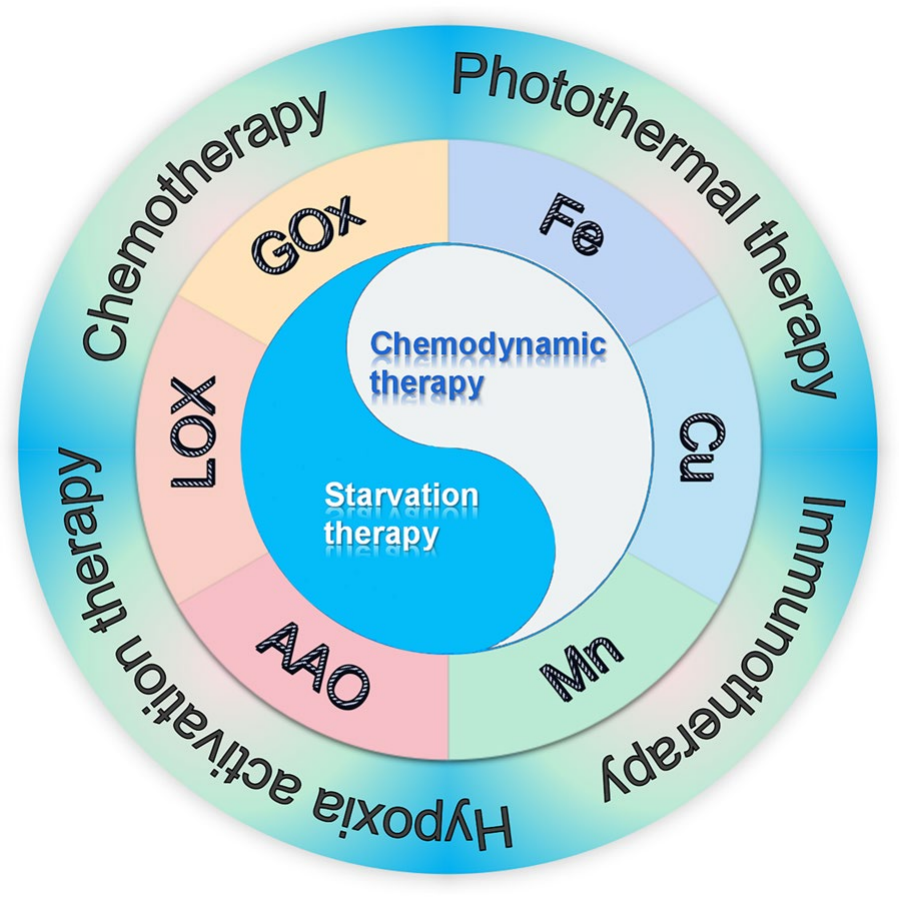
Schematic diagram of the synergistic anti-tumor effect of the "metal-oxidase" cascade catalytic system and the combination of this system with other anti-tumor therapies

This article first introduces the applications and limitations of CDT induced by Fenton/Fenton-like reaction catalyzed by iron, copper, and manganese in tumor treatment. Secondly, the applications and limitations of starvation therapy induced by intracellular metabolite oxidases (GOx, LOX, AAO) in tumor treatments are introduced. Subsequently, the synergistic effects and applications of the “metal-oxidase” cascade catalytic system formed by loading various oxidases on three metals are described in detail. Consequently, the “metal-oxidase” system and other tumor treatment methods are summarized, with an emphasis on the synergistic effect with immunotherapy, hoping to provide references for comprehensive tumor treatments (Scheme 1).

## Fenton/Fenton-like reaction catalyzed by metal-based nanoparticles

Under acidic conditions, Fe^2+^ can catalyze H_2_O_2_ to produce strong oxidizing •OH, which is called as Fenton reaction. Besides, similar chemical reactions can be catalyzed by other metal ions, known as Fenton-like reaction. The main reaction equations are described as follows:

$${\text{Fe}}^{{{2} + }} + {\text{ H}}_{{2}} {\text{O}}_{{2}} \to ^{{ }} {\text{Fe}}^{{{3} + }} + \bullet {\text{OH}} + {\text{ OH}^-}$$

$${\text{Cu}}^{ + } + {\text{ H}}_{{2}} {\text{O}}_{{2}} \to ^{{ }} {\text{Cu}}^{{{2} + }} + \bullet {\text{OH}} + {\text{ OH}^-}$$

$${\text{Mn}}^{{{2} + }} + {\text{ H}}_{{2}} {\text{O}}_{{2}} \to ^{{ }} {\text{Mn}}^{{{3} + }} + \bullet {\text{OH}} + {\text{ OH}^-}$$

$${\text{Co}}^{{{2} + }} + {\text{ H}}_{{2}} {\text{O}}_{{2}} \to ^{{ }} {\text{Co}}^{{{3} + }} + \bullet {\text{OH}} + {\text{ OH}^-}$$

$${\text{Ti}}^{{{3} + }} + {\text{ H}}_{{2}} {\text{O}}_{{2}} \to ^{{ }} {\text{Ti}}^{{{4} + }} + \bullet {\text{OH}} + {\text{ OH}^-}$$

Transition metal ions catalyze H_2_O_2_ to produce a large amount of •OH, and the weak acid TME will provide a suitable pH environment for the reaction in the meantime. However, using metal-based nanoparticles alone will encounter difficulties including: insufficient endogenous H_2_O_2_, low targeting and efficiency of drug delivery modes based on the EPR effect, low catalytic efficiency of metal ions and insufficient ROS, large therapeutic doses, potential toxicity to humans, and other issues. Therefore, various functional groups will be loaded on the metal-based nanoparticles in practical applications to make up for the above shortcomings. We introduce the concrete applications of iron, copper, and manganese in tumor treatment and the pros and cons of each metal.

### Iron and Fenton reaction

As we all know, most of the materials that induce the ferroptosis of tumor cells contain iron [21]. Iron-containing nanoparticles can specifically accumulate in tumor cells through passive or active targeting and release Fe^2+^/Fe^3+^ to catalyze the Fenton reaction. Subsequently, excessive H_2_O_2_ are converted into •OH, further inducing tumor cell ferroptosis and apoptosis [22, 23]. Also, iron is a common element in human body and has a mature storage and metabolism mechanism. Neither been injected intratumorally nor intravenously can ensure the biosafety of iron-based nanoparticles [24–26]. Therefore, CDT based on the Fenton reaction catalyzed by Fe^2+^ has broad prospects in cancer treatment.

**Fig. 1:**
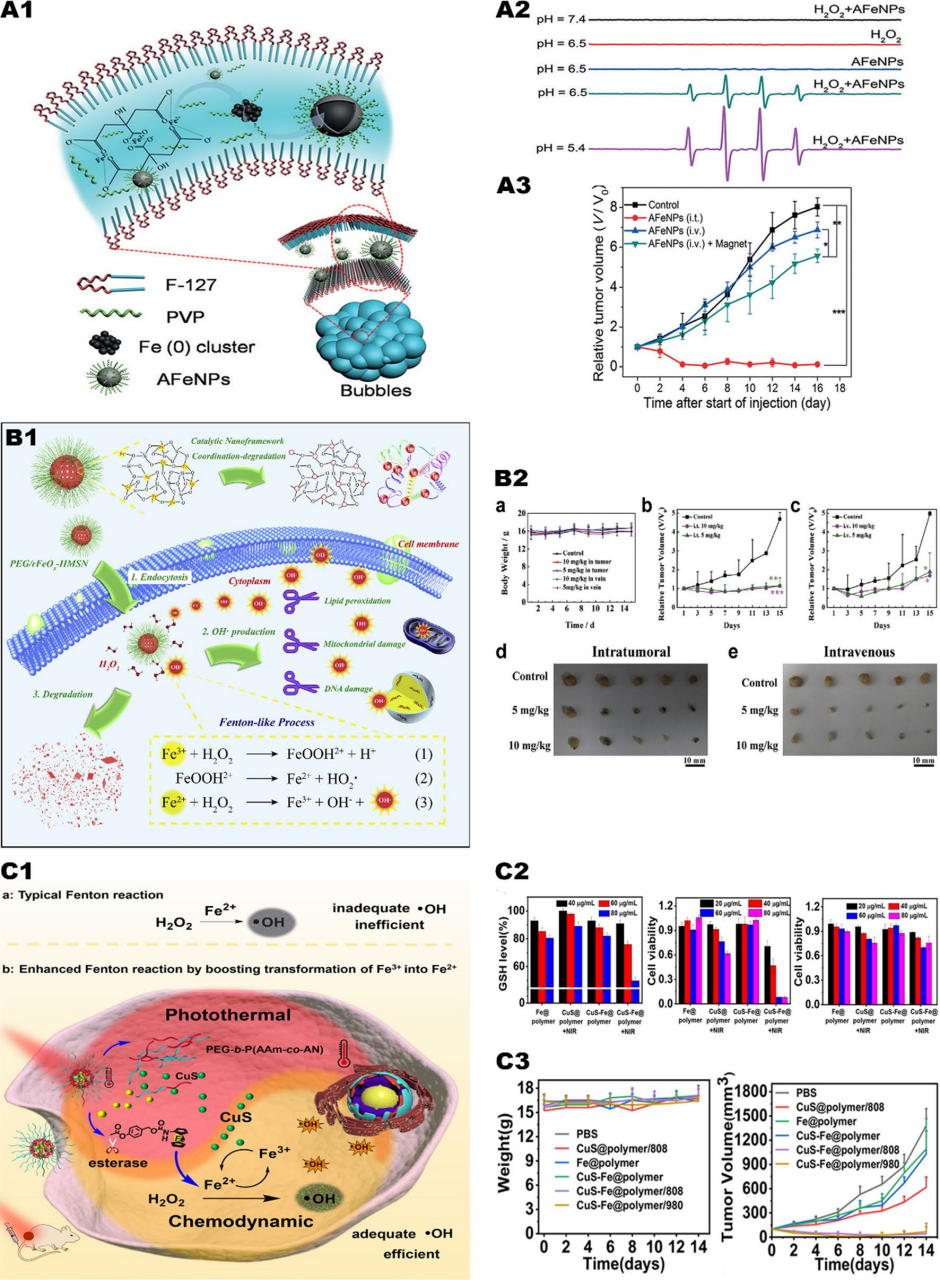
Iron-mediated Fenton reaction triggers CDT. **A1** Preparation of AFeNPs. **A2** The optimal value for AFeNPs catalyzed hydrogen peroxide decomposition was pH = 5.4. **A3** Changes in relative volume of tumors after different treatments. Reproduced with permission from Ref [28], copyright © 2016 Wiley–VCH. **B1** Schematic diagram of tumor therapeutic performance of biodegradable rFeOx-HMSN nanoparticles. **B2** (**a**) Body weight changes of 4T1 bearing mice after different treatments. Relative tumor volumes of 4T1-bearing mice administrated with varied doses of PEG/rFeOx-HMSN nanocatalyst (**b**) intratumorally and (**c**) intravenously. Digital photographs of dissected tumors after the therapeutic process from (**d**) intratumoral and (**e**) intravenous groups. Reproduced with permission from Ref [29], copyright © 2018 Elsevier. **C1** Schematic of CuS-Fe boosting transformation of Fe (III) into Fe (II) for highly improving CDT. **C2** GSH level in HeLa cells after difffferent treatments; Cell viability in HeLa cells and NIH3T3 cells after different treatments. **C3** Changes in body weight of mice and tumor volume during different treatments. Reproduced with permission from Ref [30], copyright © 2019 American Chemical Society

Fu et al. [27] proposed a simple in-situ oxidative and reductive method, which uses mesoporous silica nanomaterials loaded with iron oxide (FeOx-MSN). FeOx-MSN can enter the acid lysosomes of tumor cells and catalyze the decomposition of H_2_O_2_ to produce a large amount of •OH. In vitro experiments, after adding exogenous H_2_O_2_, the toxicity of nanoparticles was greatly enhanced, and the viability of cancer cells incubated with 100 µg/mL FeOx-MSNs and 100 µM H_2_O_2_ dropped from 96.2% to 40.6%. Zhang et al. [28]. exploited an amorphous iron nanoparticle via a mechanism of using endogenous H_2_O_2_ to generate ROS in cancer cells (Fig. 1,A1). The nanoparticles ionize in the acidic TME and release Fe^2+^, which further induces the local Fenton response of the tumor (Fig. 1, A2). In an in vivo study, 16 days after the injection of nanoparticles into the tumor, tumor growth was completely inhibited (Fig. 1, A3). In a similar study, Wang et al. [29]. reported the application of iron-based silica nanoparticles (rFeOx-HMSN) as a Fenton reagent in tumor treatment in vivo and in vitro (Fig. 1, B1). In a protein-rich tissue environment, nanoparticles are easy to collapse with release of Fe^2+^ and Fe^3+^ and destroy cancer cells through the Fenton reaction. Under the conditions of 50 µg/mL rFeOx-HMSN and 50 µM exogenous H_2_O_2_ (pH 6.0), the survival rate of cancer cells is reduced to less than 30%. In in vivo studies, tumor growth was inhibited after 15 days of intratumor injection and intravenous injection of nanoparticles (Fig. 1, B2). However, Fe^2+^ with high catalytic activity is easily oxidized to Fe^3+^ with low catalytic activity, which greatly reduces the efficiency of the Fenton reaction. If Fe^3+^ can be quickly converted into Fe^2+^, it will undoubtedly greatly accelerate the Fenton reaction. Nie et al. [30] constructed a prodrug containing CuS and Fe nanoparticles (Fig. 1, C1), the surface of which was coated with a temperature-sensitive polymer. By irradiating tumor tissue with near-infrared light, CuS can generate a lot of heat to promote the thermal ablation of cancer cells, and release Fe^2+^ to activate the Fenton reaction to form •OH and Fe^3+^. On the other hand, CuS can promote the regeneration of Fe^3+^ to Fe^2+^, thereby maintaining the Fe^2+^ concentration in tumor cells. Both in vitro and in vivo experiments have proved that under near-infrared light irradiation, CuS-Fe nanoparticles can significantly inhibit tumor growth and have reliable biosafety (Fig. 1, C2, C3).

Iron-based nanoparticles have catalytic activity, active oxygen induction, and drug loading capabilities, which provide the possibility for effective tumor synergistic therapy. Therefore, our research group published an article based on Fe^2+^ catalyzed Fenton reaction and photodynamic therapy in 2020 [31]. We synthesized iron-tannic acid composite nanoparticles (HFe-TA) with a fine hollow microstructure and loaded the photosensitizer indocyanine green (ICG) for synergistic tumor treatment. Tannic acid (TA) is one of the natural polyphenols approved by the U.S. Food and Drug Administration (FDA). It can coordinate with metal ions such as Fe^3+^ to form metal-phenol net-works (MPN). TA has high reducibility in an acidic environment and can reduce Fe^3+^ to Fe^2+^ with a better catalytic effect, but it has no reducibility in a neutral environment. ICG is an efficient photosensitizer that can convert O_2_ into active oxygen under the irradiation of near-infrared light. ICG@HFe-TA decomposes and releases Fe^3+^, TA, and ICG molecules in the acidic TME. Fe^3+^ with low catalytic activity can be reduced by TA to Fe^2+^ with high catalytic activity, which can promote the Fenton reaction more efficiently. More importantly, O_2_ is generated during the conversion of Fe^3+^ to Fe^2+^, and the ROS induced by ICG increases significantly (1, 2). At the same time, ICG@HFe-TA will not generate reactive oxygen species in normal tissues with a neutral pH, so it has better targeting function and biosafety. Both in vivo and in vitro experiments prove that ICG@HFe-TA has a good tumor-inhibiting effect.

1 $${\text{Fe}}^{{{3} + }} + {\text{ H}}_{{2}} {\text{O}}_{{2}} \to ^{{ }} {\text{Fe}}^{{{2} + }} + \, \bullet {\text{OOH }} + {\text{ H}}^{ + }$$

2 $${\text{Fe}}^{{{3} + }} + \, \bullet {\text{OOH}}\to ^{{ }} {\text{Fe}}^{{{2} + }} + {\text{ O}}_{{2}} + {\text{ H}}^{ + }$$

### Copper and Fenton-like reaction

Fe^2+^ catalyzes the Fenton reaction at a low rate (k = 63–76 M^−1^ s^−1^). The conversion from Fe^3+^ to Fe^2+^ is slow (k = 0.001–0.01 M^−1^ s^−1^), and strongly depends on the acidic environment (pH 2 ~ 4) [32]. Cu^+^/Cu^2+^ has a higher catalytic rate (k = 1.0 × 10^4^ M^−1^ s^−1^), and the conversion from Cu^2+^ to Cu^+^ is also very fast (k = 4.6 × 10^2^ M^−1^ s^−1^), also performing well in a wide pH [33–35]. The level of glutathione (GSH) in tumor tissue is high. GSH can counteract the strong oxidation of •OH and weaken the effect of the Fenton reaction [34]. Cu^2+^ can undergo redox reaction with GSH to generate Cu^+^ and oxidized glutathione (GSSG). Cu^2+^ consumes GSH on the one hand, and generates Cu^+^ on the other hand, which improves the catalytic efficiency of the Fenton reaction (Cu^2+^  + GSH $\to ^{{ }}$ Cu^+^  + GSSG). Therefore, Cu^2+^ can inhibit tumors more efficiently than Fe^2+^.

**Fig. 2:**
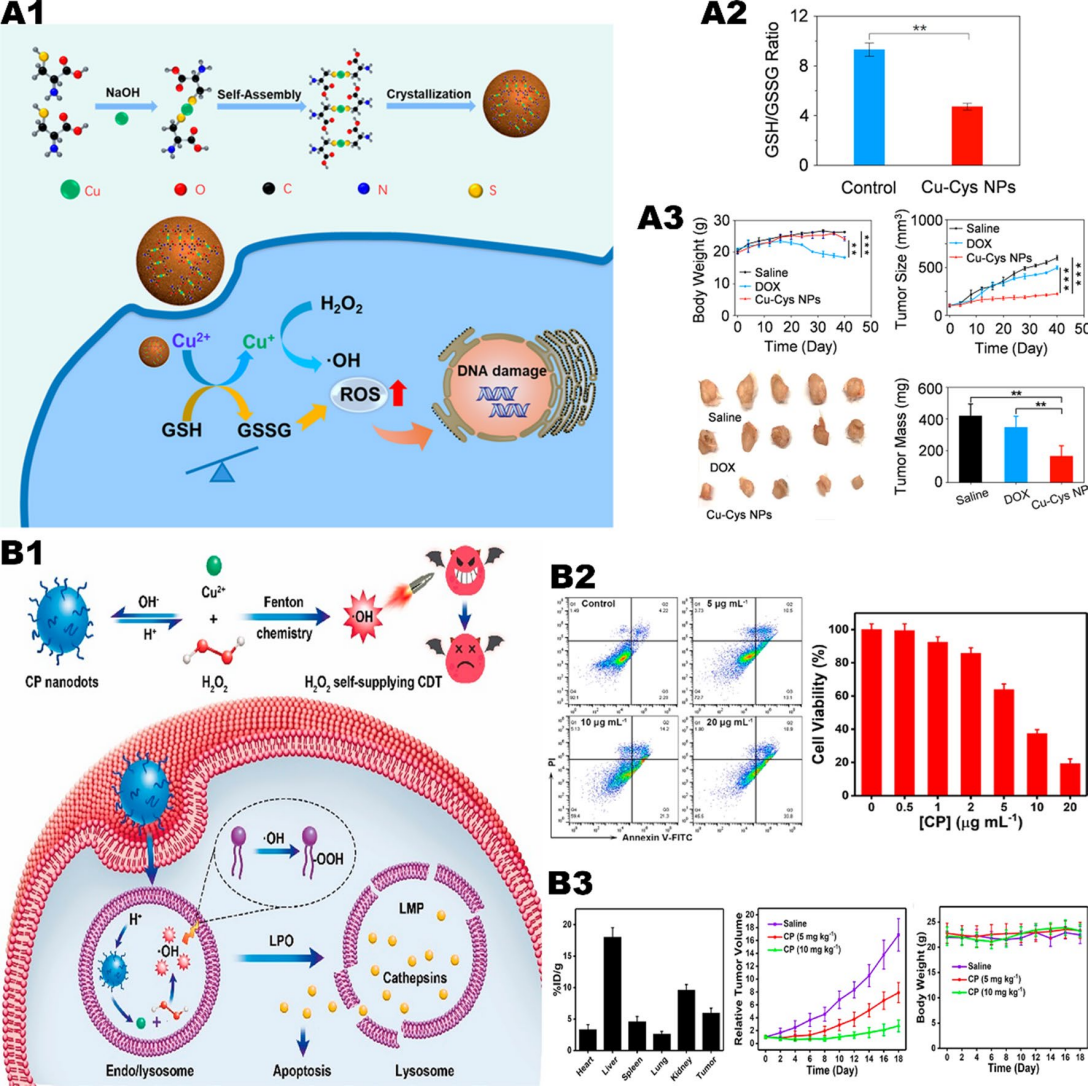
Copper element mediates Fenton reaction to trigger CDT. **A1** Schematic of the Cu-Cys NPs synthetic process and the copper-containing nanoformulation mediated CDT. **A2** GSH/GSSG ratio in MCF-7R cells before and after treatment with 100 μg/mL of Cu-Cys NPs. **A3** Changes in body weight of mice and tumor volume during different treatments. Reproduced with permission from Ref [34], copyright © 2018 American Chemical Society. **B1** Formation of CP nanodots for H_2_O_2_ self-supplying CDT. **B2** Flow cytometry analysis of apoptosis in U87MG cells treated with CP nanodots for 12 h and CDT potency of CP nanodots after 24 h of incubation. **B3** Biodistribution of Cu in major organs and tumor of U87MG tumor-bearing mice at 24 h post i.v. injection with CP nanodots and changes in body weight of mice and tumor volume during different treatments. Reproduced with permission from Ref [36], copyright © 2019 American Chemical Society

Compared with monochemotherapy, CDT induced by Cu-based nanoplatform may have more advantages. Ma et al. [34] constructed copper-amino acid sulfhydryl nanoparticles (Cu-Cys-NPs) (Fig. 2, A1). When the nanoparticles are internalized by breast cancer cells, Cu^2+^ reacts with local GSH and induces GSH depletion (Fig. 2, A2), with Cu^2+^ been reduced to Cu^+^. Subsequently, the generated Cu^+^ reacts with local H_2_O_2_ to generate highly toxic •OH, leading to tumor cell apoptosis. Due to the appropriate pH value and high concentrations of GSH and H_2_O_2_ in tumor cells, in vitro experiments showed that Cu-Cys-NPs performed high cytotoxicity in tumor cells. The inhibitory rate of 5 mg/kg Cu-Cys-NPs on tumors (72.3%) is higher than that of the chemotherapy drug doxorubicin (DOX) (only 17.1%) in vivo. Compared with the same dose of DOX, Cu-Cys-NPs have satisfactory antitumor effect in drug-resistant breast cancer (Fig. 2, A3).

Similarly, the Cu^+^/Cu^2+^ catalytic system also faces the problem of insufficient endogenous H_2_O_2_, which undoubtedly reduces the ability to generate ROS to a certain extent. If an excessive amount of H_2_O_2_ is carried on the metal nano-platform, then the problem can be solved. Lin et al. [36]. prepared copper peroxide nanoparticles (CP) through the coordination of H_2_O_2_ and Cu^2+^ with the assistance of hydroxide ions (Fig. 2, B1). CP NPs enters the lysosome through tumor cell endocytosis, and the nanoparticles dissociate rapidly in an acidic environment, releasing Cu^2+^ and H_2_O_2_, and catalyzing the Fenton-like reaction. The resulting •OH leads to lysosomal membrane lipid peroxidation and cell death through lysosomal-related pathways. In addition to the pH dependence, micro-size CP NPs exhibit high tumor aggregation after intravenous administration, thereby effectively inhibiting tumor growth in vivo with minimal side effects. When U87MG cells were treated with 20 ug/mL CP NPs for 24 h, the cell viability was significantly reduced to below 20% (Fig. 2, B2). At the same time, CP NPs show less toxicity on normal cells, which have also been proved in vivo (Fig. 2, B3).

On the one hand, copper ions can inhibit tumors through the Fenton-like reaction, but on the other hand, excessive copper ions can also cause greater damage to the body, such as damage to liver and gallbladder function, inducing hepatolenticular degeneration and cardiovascular disease, and leading to higher cytotoxicity and carcinogenesis [37]. Therefore, when using copper-based nanoparticles, more attention needs to be paid to their targeting function and biosafety.

### Manganese and Fenton-like reaction

Comparative analysis shows that pH 2 ~ 4 is the optimal condition for the Fenton reaction catalyzed by Fe^2+^, but there is no catalytic activity at pH 5 ~ 8. The difference is that Mn^2+^ exhibits effective catalytic activity in the entire pH range, and has the best catalytic activity at pH 5 [38]. Similar to Cu^2+^, MnO_2_ can also consume GSH and produce Mn^2+^ with higher catalytic efficiency. More importantly, recent studies have shown that Mn^2+^ can activate the cGAS-STING pathway and effectively promote immunity, which is a kind of nanocarrier that has been seriously “ignored”.

**Fig. 3:**
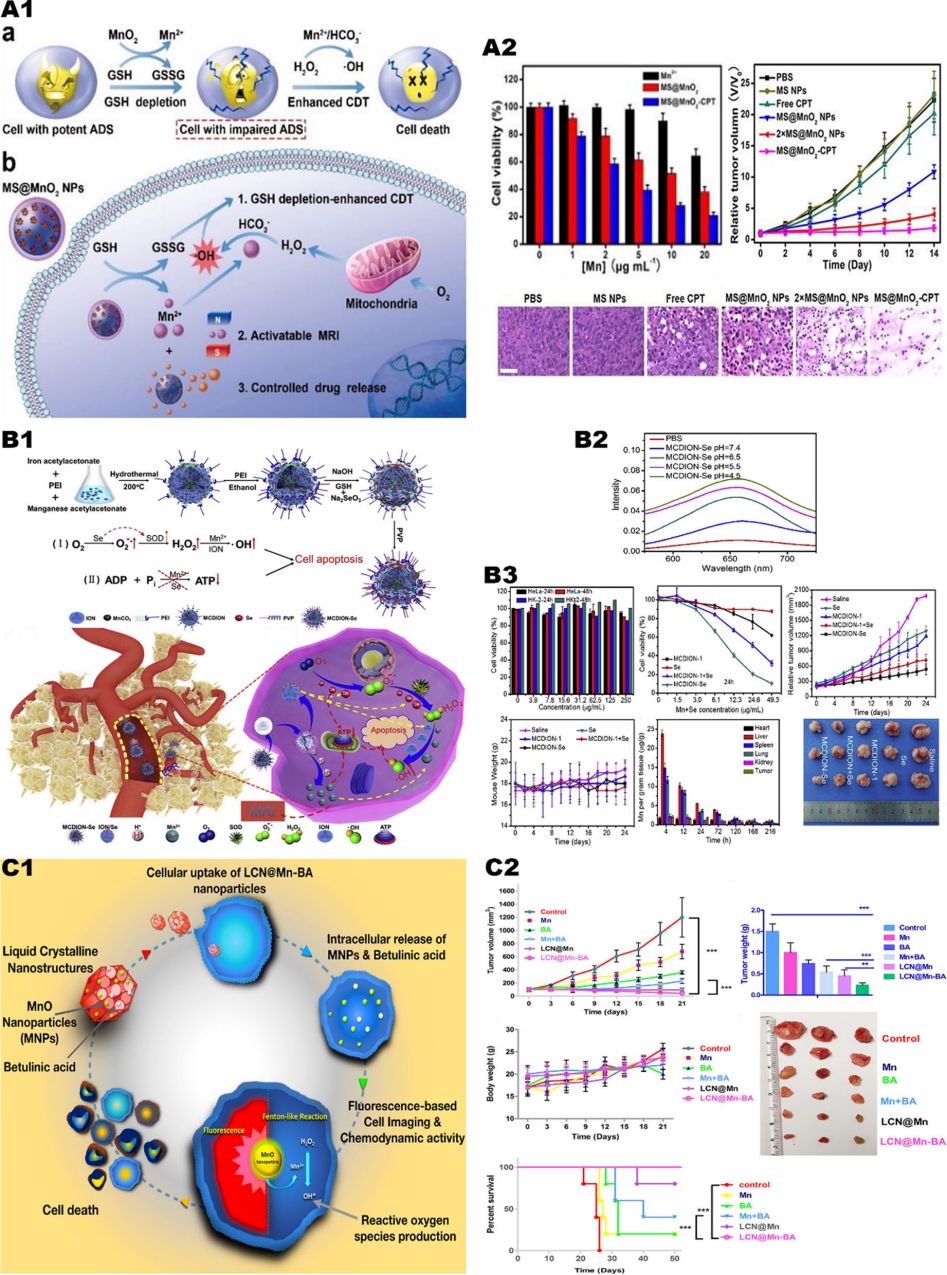
Manganese mediates the Fenton reaction to trigger CDT. **A1** Schematic illustrating the application of MS@MnO_2_ NPs for MRI-monitored chemo-chemodynamic combination therapy. **A2** Viability of U87MG cells after 24 h of different treatments. And viability of U87MG cells after 24 h of different treatments. And H&E-stained images of tumour sections from different groups. Reproduced with permission from Ref [39], copyright © 2018 WILEY–VCH. **B1** Illustration of the synthetic process of MCDION-Se. And the cascade reaction of MCDION-Se in the intracellular environment. **B2** The higher the degree of acidity the stronger the catalytic reaction. **B3** Relevant in vitro experiments and in vivo experiments demonstrated the efficacy and safety of MCDION-Se. Reproduced with permission from Ref [40], copyright © 2019 Elsevier. **C1** Schematic diagram and efficacy of LCN@Mn-BA nanoparticles for anti-tumor. **C2** In vivo experiments in mice demonstrated LCN@Mn-BA can effectively inhibit tumor growth with good biosafety. Reproduced with permission from Ref [38], copyright © 2020 Elsevier

Lin et al. [39] reported MnO_2_ coated mesoporous silica nanoparticles MSN@MnO_2_ NPs (Fig. 3, A1). After being endocytosed by tumor cells, it undergoes a redox reaction with GSH to generate glutathione disulfide and Mn^2+^. The generated Mn^2+^ induces a Fenton-like reaction, which catalyzes the generation of endogenous H_2_O_2_ and •OH to kill tumor cells. According to the results of in vitro experiments, MSN@MnO_2_ has a better anti-cancer effect than Mn^2+^ alone (Fig. 3, A2).

MnO_2_ can consume GSH, reduce the reducing substances in tumor cells, and expand the anti-tumor effect of ROS. However, MnO_2_ will consume endogenous H_2_O_2_, leading to insufficient raw materials for the Fenton-like reaction and reducing the antitumor effect. Therefore, when using MnO_2_, a group with the function of generating H_2_O_2_ can be loaded to further enhance the anti-tumor effect of CDT. Xiao et al. [40] constructed nano-selenium-coated manganese carbonate nanoparticles MCDION-Se (Fig. 3, B1). MCDION-Se can release a large amount of Mn^2+^ ions, and effectively induce cancer cell apoptosis through Fenton-like reaction (Fig. 3, B2). In addition, the nano-selenium coated on the surface of MCDION-Se can also significantly activate superoxide dismutase (SOD) in tumor tissues and promote the generation of superoxide anion free radicals (SOARs) in tumor tissues. Subsequently, SOD catalyzes SOARs to produce excessive H_2_O_2_, which further improves the efficiency of chemotherapy. At the same time, experiments show that nano-selenium and Mn^2+^ ions can inhibit the production of adenosine triphosphate (ATP), thereby starving tumor cells. (Fig. 3, B3).

Mn^2+^ can also be used in combination with chemotherapy drugs. Urandur et al. [38] reported a liquid crystal nanoparticle (LCN@Mn-BA) loaded with MnO_2_ and betulinic acid (BA) (Fig. 3, C1). In in vivo experiments, compared with the blank control group, the tumor growth inhibition index of LCN@Mn-BA reached 96.5%, achieving significant tumor ablation (Fig. 3, C2).

In addition, Lv et al. [41] found that exogenous addition of Mn^2+^ can effectively activate the cGAS-STING pathway of human or mouse cells, further enhancing the immune surveillance and immune clearance of tumor cells. In conclusion, Mn^2+^ can catalyze Fenton-like reaction in a wide range of pH. Unlike other metal ions, Mn^2+^ can also activate cGAS-STING pathway and further promote tumor immunity, finally achieving antitumor effect from two aspects. Although the current efficacy of tumor immunotherapy fails to meet our demand, with the immunotherapy being refined, more patients will surely benefit from it. Therefore, we reasonably infer that manganese is likely to become one of the breakthroughs in immunotherapy, and it is a promising metal-based nanocarrier.

### Recent advances in transition metals catalyzed Fenton/Fenton-like reaction

Iron, copper and manganese are the most common catalysts for Fenton/Fenton-like reactions. Surely, in addition to the above three metals, there are many other transition metals with similar catalytic capabilities, such as Ti, Co, and Mo. Liang et al. [42] constructed a defect-rich Ti-based metal–organic framework (MOF) (D-MOF(Ti)). Benefiting from the components of Ti^3+^ ions. D-MOF(Ti) have been illustrated to have excellent potentials in Fenton-like activity to enable CDT.

Fu et al. [43] synthesized PEGylated CoFe_2_O_4_ nanoflowers (CFP) for autologous sonodynamic therapy (SDT) and CDT with optimization of robust immune response. The CFP occupying multivalent elements (Co^2+/3+^, Fe^2+/3+^) manifested strong Fenton-like and catalase-like activity. The bimetallic carboxylic Li/Co-MOFs synthesized by Li et al. [44] are possessed with a tetrahedral coordination metal center, showing efficient Fenton-like reaction catalytic capacity. Li et al. [45] found that the formation of the Tris-Co^2+^ complex could reduce the oxidation–reduction potential of the Co^3+^/Co^2+^, facilitating the conversion of the Tris-Co^3+^ complex into the Tris-Co^2+^ complex, accelerating the rate of Fenton-like reaction in the system.

Being a co-catalyst in the Fe^2+^/H_2_O_2_ Fenton system, MoS_2_ can greatly facilitate the Fe^3+^/Fe^2+^ cycle reaction by the exposed Mo^4+^ active sites, which significantly improves the H_2_O_2_ decomposition efficiency for the •OH production [46].

## Oxidase-mediated starvation therapy and oxidative therapy

The oxidation catalytic process mediated by GOx can effectively consume glucose and oxygen, producing a large amount of H_2_O_2_ and acidic substances simultaneously (1), which can provide sufficient substrate and suitable reaction environment for the Fenton/Fenton-like reaction [7–9]. Decreased glucose levels in tumors can effectively block the energy supply of tumor cells to “starve” tumor cells, which is called as tumor starvation therapy [11]. In addition, the increasing acidity of the TME can be used to activate pH-sensitive drug nanovectors to continuously release antitumor drugs [47]. At the same time, the excessive H_2_O_2_ produced in tumor cells can significantly increase the oxidative stress in the tumor, which further leads to cell necrosis [48, 49]. Similarly, LOX [50] and AAO [51] can also catalyze the formation of acidic substances and H_2_O_2_ from lactic acid and amino acids, which can further generate •OH and combine with starvation therapy to exert antitumor effects (4, 5). The synergistic effect of the above-mentioned intracellular metabolite oxidases and transition metal can maximize the effect of CDT, and the combination with starvation therapy further amplifies the antitumor effect. Here, we briefly introduce the applications of GOx, LOX, and AAO in tumor treatment and their respective advantages and disadvantages.

3 $${\text{Glucose }} + {\text{ O}}_{{2}} \mathop{\longrightarrow}\limits^{{\text{ GOX }}}{\text{ Gluconic acid }} + {\text{ H}}_{{2}} {\text{O}}_{{2}}$$

4 $${\text{Lactic acid }} + {\text{ O}}_{{2}} \mathop{\longrightarrow}\limits^{{\text{ LOX }}}{\text{ Pyruvic acid }} + {\text{ H}}_{{2}} {\text{O}}_{{2}}$$

5 $${\text{Amino acid }} + {\text{ O}}_{{2}} \mathop{\longrightarrow}\limits^{{{\text{AAO}}}}{\text{Ketoic acid }} + {\text{ Ammonia }} + {\text{ H}}_{{2}} {\text{O}}_{{2}}$$

### Glucose oxidase in antitumor therapy

Unlike normal cells, tumor cells prefer inefficient anaerobic glycolysis rather than oxidative phosphorylation, which means tumor cells have to increase their glucose uptake to acquire enough nutrients for proliferation [52]. This characteristic provides us with an opportunity to speculate tumor growth by measuring the glucose concentration in the solid tumor. Furthermore, based on the stoichiometry of the GOx catalytic reaction, tumor glucose intake can be easily and quickly calculated via measuring oxygen consumption or the amount of H_2_O_2_, further contributing to infer tumor progression [9].

Besides tumor diagnosis, GOx-mediated glucose consumption provides a non-invasive strategy [48, 53] for tumor starvation therapy. Nevertheless, the application of natural GOx is limited due to its poor stability, short half-life, immunogenicity. More importantly, for the reason that both glucose and oxygen are prevalent in vivo, intravenous administration would cause the scattering of H_2_O_2_ in human body, resulting in severe systemic adverse effects. Therefore, it is of great significance to design appropriate nanovectors to protect GOx. Zhao et al. [54] have synthesized the GOx − poly (FBMA-co-OEGMA) nanogels (NGs) for synergistic tumor starvation and oxidation therapy. Fan et al. [55] have used hollow mesoporous organosilica nanoparticle (HMON) as a vector and loaded with GOx for tumor starvation therapy. All the nanomaterials mentioned above adopt instinct vectors to package GOx, not only increasing the circulation time and the stability of the drugs, but also decreasing the systemic adverse effects, greatly enhancing the efficacy and biosafety of the drugs.

Moreover, the oxygen consumption of GOx aggravates the degree of hypoxia in the TME, which also obviously limits the GOx catalytic reactions in turn. Dinda et al. [11] constructed self-assembled vesicles of trimesic-acid-based biotinylated amphiphile (TMB) with GOx wrapped in it, which mediated targeted tumor starvation therapy. In vitro experiments showed that GOx-loaded TMB vesicles were six times more lethal on tumor cells than on normal cells. However, there would be a reduction on tumor killing effects When the GOx concentration is 500 µg/mL, probably because of the limited catalytic responses of GOx in the hypoxia environment. Therefore, combining GOx with drugs facilitating oxygen production to form a synergistic therapy becomes the key to solve the problem. It is proved that peroxidase (e.g. catalase) [56] or peroxide inorganic catalyst (e.g. Prussian blue nanoparticles) [53] could availably stimulate the disproportionation reaction of H_2_O_2_ to generate oxygen. On the one hand, combining GOx with those compounds can reach the aim of accelerating glucose consumption. On the other hand, it provides new sights for designing novel collaborative treatments based on the mechanism.

**Fig. 4:**
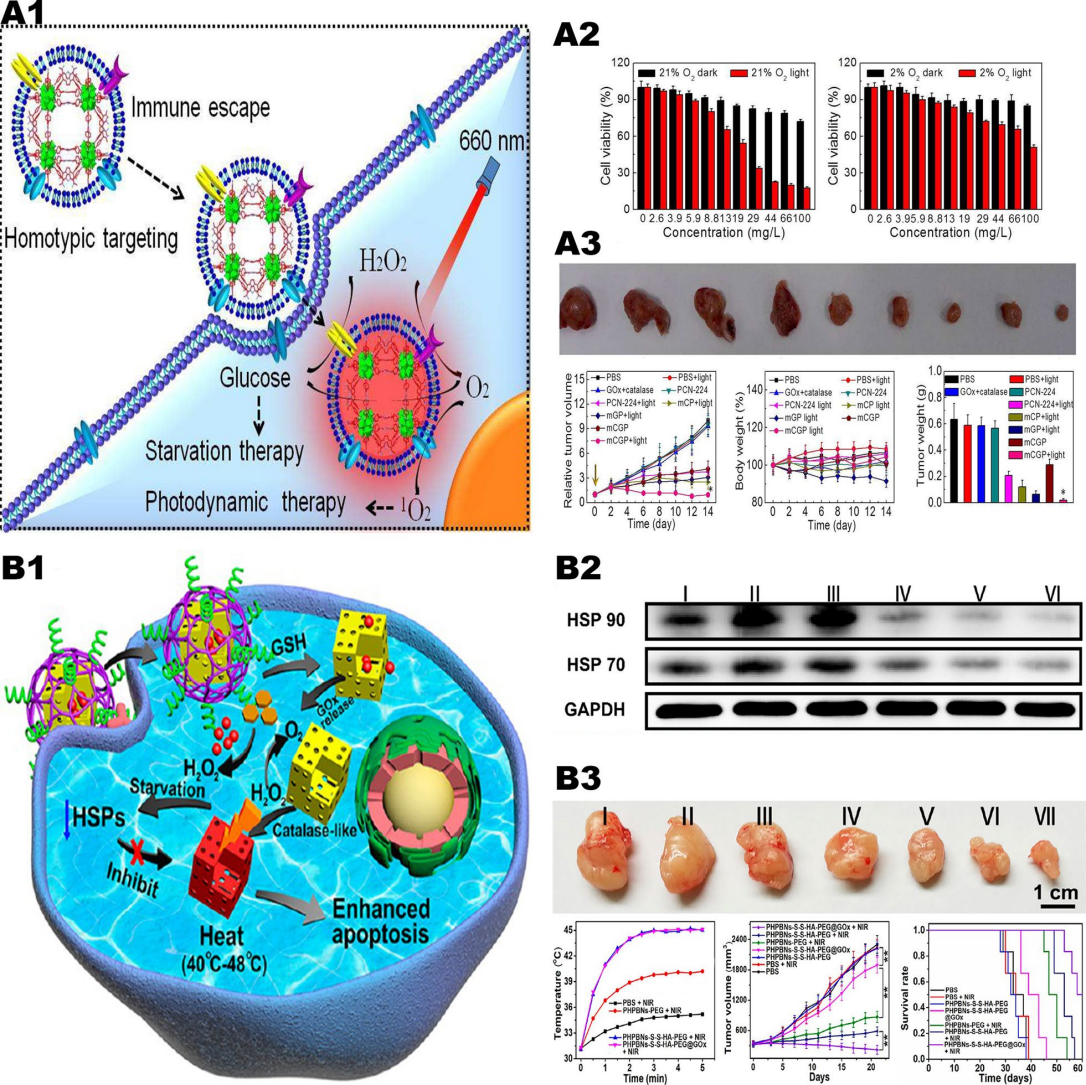
Glucose oxidase-mediated starvation therapy. **A1** Schematic illustration of the mCGP nanoparticles for cancer targeting starvation therapy and PDT. **A2** The dark and light toxicities of mCGP against 4T1 cells by MTT assay under 21% O_2_ or 2% O_2_. **A3** The anticancer efficiency of mCGP in vivo. Reproduced with permission from Ref [56], copyright © 2017, American Chemical Society. **B1** Illustration of GOx-induced starvation for enhanced low-temperature PTT in a hypoxic TME. **B2** Representative Western blotting of HSP 90 and HSP 70 expression under different conditions. **B3** In vivo experiments in mice, the nanoparticles were able to effectively inhibit tumors and prolong mouse survival time. Reproduced with permission from Ref [53], copyright © 2018, American Chemical Society

The researchers formed a porous coordination network framework [56] by embedding GOx and catalase into cancer cell membranes to realize synergistic therapy (Fig. 4, A1). Due to the bionic surface this frame owns, it can craftily escape from the elimination of immune system and manifest as an isotypic targeting behavior, contributing to targeting on tumor cells targeting and accumulating in tumor issues as a retentate. Once entered tumor cells, the framework catalyzes endogenous H_2_O_2_ to generate oxygen, which can not only regulate the hypoxic TME, but also offset the oxygen consumed by glucose decomposition. Furthermore, the product oxygen can also enhance the production of cytotoxic reactive oxygen under light, further improving the synergistic action of starvation therapy and photodynamic synergy therapy (Fig. 4, A2, A3). This synergistic therapy can also be combined with PTT, and a GOx-loaded Prussian blue nanoparticle has been synthesized by researchers (Fig. 4, B1) [53]. Via the endocytosis of tumor cells, GOx can be released and consumes glucose and oxygen within the tumor to generate excessive H_2_O_2_. While Prussian blue nanoparticles were able to decompose H_2_O_2_ into oxygen, thus they perform an enhancement of the effect of starvation therapy in hypoxic tumor tissue. More importantly, nanoparticles can inhibit the expression of heat shock protein after PTT, thereby reducing the tolerance of tumor tissue to the low-temperature PTT mediated by Prussian blue particles. Experiments in tumor-bearing nude mice showed that the inhibiting tumor rate in the combination treatment group of tumor starvation therapy with low-temperature PTT was about 15 times greater than that in the negative control group (Fig. 4, B2).

On the other hand, the hypoxia environment resulted from GOx-mediated oxygen consumption can also be well exploited, for the reason that increased hypoxia levels in the TME can activate hypoxia-activated prodrugs, such as banoxantrone dihydrochloride (AQ4N) and tirapazamine (TPZ) [48, 57]. And the generated glucose acid also helps to acidify the TME, triggering pH-responsive drug release [47]. Furthermore, the excessively generated H_2_O_2_ have been proved to not only significantly increase tumor oxidative stress, but also attack tumor cells in the form of •OH converted from CDT mediated by Fenton reaction. And the details would be comprehensively introduced in our next chapter [58, 59].

### Lactate oxidase in antitumor therapy

Tumor cells undergoing aerobic glycolysis require a large amount of glucose and generate excessive lactate in the TME. To get enough nutrients and ATP to meet the demand of proliferation, tumor cells have to increase the uptake of glucose and accelerate the glycolysis rate, resulting in the accumulation of lactate [60]. The excessive lactate has been proved to effectively lead to the acidification of the TME. Recently, increasing studies suggest that the accumulated lactate in the TME as well as the lactate-induced acidic environment can resist on tumor immunity by negative regulating infiltrating immune cells [61–63]. At the same time, lactate can act as a signaling molecule to promote tumor angiogenesis [64], regulate cell cycle and inhibit tumor apoptosis pathway, facilitating tumor proliferation and invasion [65, 66]. Of note, recent studies have reported that lactate can provide energy for tumor tissues [67, 68]. Meanwhile, the acidification of the TME weaken the efficacy of exogenous alkaline antitumor drugs [69, 70]. These factors undoubtedly pose new challenges to the treatment of tumors. It has been reported that inhibiting lactate dehydrogenase to decrease the lactate level can serve as a novel tumor treatment [71, 72]. Currently, the application of LOX is relatively mature for tumor treatment. On the one hand, LOX can reduce the degree of acidification while consuming lactate in the TME, activating tumor immunity response and further remodeling a protective TME. On the other hand, the excessively generated H_2_O_2_ can not only directly kill tumor cells via its strong oxidative ability, but combine with multiple antitumor therapies to achieve synergistic effects.

**Fig. 5:**
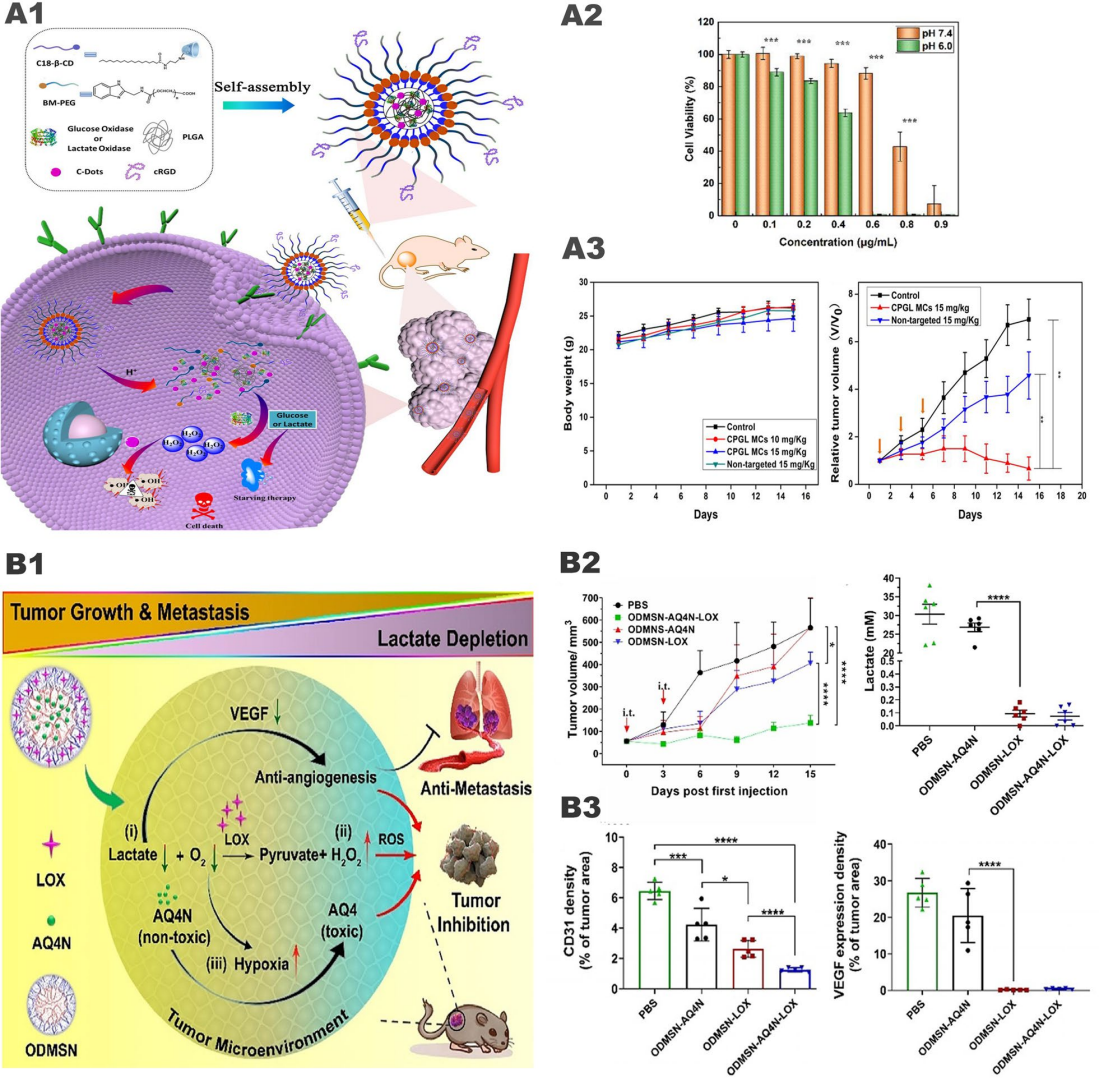
Lactate oxidase-mediated starvation therapy. **A1** Schematic diagram of tumor-targeted CPGL micelles as the combination of starving and catalytic therapy basing on the generation of highly toxic ▪OH. **A2** In vitro cytotoxicity of B16 cells after treated with CPGL micelles (0–0.9 μg/mL) for 24 h under different conditions (pH 7.4 and pH 6.0). **A3** Monitoring body weight of mice every 2 days. And tumor volumes of B16 bearing mice after injections intravenously. The yellow arrow showed the injection time. Reproduced with permission from Ref [73], copyright © 2020, American Chemical Society. **B1** Illustration of lactate-depletion-enabled TME adjustment and combinational cancer treatment strategy. **B2** Tumor growth curves of mice after different treatments. And lactate concentration in 4T1 tumors at 48 h post-injections. **B3** Numbers of blood vessels/field and percentages of VEGF-stained area were analyzed in tumor sections. Reproduced with permission from Ref [50], copyright © 2020 WILEY–VCH

Lactic acid depletion is an effective strategy for TME regulation to expand the function of other anti-tumor therapies. Wang et al. [73] synthesized supramolecular micelles with tumor targeting and pH sensitivity, which consume glucose and lactic acid in tumors by loading GOx and LOX and generate H_2_O_2_ and acidic substances at the same time. And through the loaded C-dot nanoenzyme (with peroxidase activity to reduce H_2_O_2_ to highly toxic •OH (Fig. 5, A1). After intravenous injection, the targeting peptide and specific pH-sensitive micelles can ensure the delivery of the load to the tumor site and specifically generate •OH at the tumor site, thereby reducing side effects. At the same time, it has a good anti-tumor effect and minimal side effects (Fig. 5, A2, A3). Tumor-derived lactate plays the pivotal role in functional macrophages from the M1 to M2 phenotype, which in turn promotes tumor growth [74]. M1 macrophages express high levels of interleukin-12 (IL-12) and IL-23, inducing inflammatory responses and promoting immunity responses to suppress tumor growth. Conversely, M2 macrophages express high levels of IL-10 and scavenger receptor, can promote tumor angiogenesis and immunosuppression, facilitating tumor angiogenesis and immunologic suppression [75]. Liao et al. [76] designed methylcellulose (MC) hydrogel incorporating LOX (MC-LOX) for repolarization of M2 to M1 macrophages by consuming lactic acid in the TME.

Elevated extracellular lactate level induces the secretion of vascular endothelial growth factor (VEGF), further leading to tumor angiogenesis [77]. Tang et al. [50] reported a one-pot synthesis of openwork@ dendritic mesoporous silica nanoparticles (ODMSNs) loaded with LOX (Fig. 5, B1), consuming more than 99.9% lactate in the TME to suppress tumor angiogenesis (Fig. 5, B2-B2). Similar to GOx, the lactate oxidation reaction catalyzed by LOX is greatly limited in a hypoxic environment. Tang et al. [78] creatively proposed to jointly assemble LOX, catalase and VEGF siRNA (siVEGF) for the synergistic inhibition of tumor proliferation and angiogenesis. Catalase catalyzes H_2_O_2_ to generate oxygen, and further activates LOX to achieve lactate depletion in the TME. The combination of LOX and siVEGF can effectively inhibit angiogenesis and 4T1 cell migration in vitro, with satisfactory inhibition of anti-antitumor and anti-metastatic capacity in vivo.

Tseng et al. [79] adopted a new antitumor idea, using LOX to activate viral therapy. They reported a low irritation vector self-assembled from hyaluronic acid (HA) and 6-(2-nitroimidazole) hexylamine for local delivery of recombinant adeno-associated virus serotype 2 (AAV2). The carrier is loaded with LOX, which can accumulate in tumor tissues with high lactic acid content. Subsequently, the LOX in the carrier oxidizes the lactic acid to pyruvate, and at the same time, the oxygen partial pressure is also reduced, biological reduction and electrostatic separation of the carrier and AAV2. Subsequently, AAV2 encodes transduced cells to express the light-sensitive KillerRed protein, which has been proven to effectively produce ROS under light, damage DNA, and induce cell apoptosis. In vivo experiments have confirmed that its anti-tumor effect is obvious, and it has biological safety at the same time.

### Amino acid oxidase in antitumor therapy

AAO is divided into l-amino acid oxidase (LAAO) and d-amino acid oxidase (DAAO). Both of them are able to oxidize the corresponding amino acids and generate strong oxidizing H_2_O_2_, further inducing synergy between tumor starvation therapy and oxidative therapy [10, 80, 81]. However, natural AAO is extremely unstable in vivo, which impairs its clinical effects. The oxygen concentration in solid tumors is another factor for the antitumor effects of both GOx and LOX. Hence, it is not a wise option to directly use natural AAO in tumor treatment. Improving AAO through protein engineering technology or loading AAO on suitable nanovectors could greatly increase their antitumor efficacy.

Rosini et al. [82] used protein engineering technology to design a variant enzyme of DAAO that can function well at low O_2_ and D-alanine concentrations. And they further improved the stability and pronged the half-life of DAAO through polyacetylation (PEG-DAAO) to enhance the EPR effect. PEG-DAAO have been observed to show strong catalytic activity against D-alanine, D-valine and D-tryptophan, generating massive H_2_O_2_ with strong oxidizability to kill tumor cells. In vitro experiments demonstrated that the extremely low PEG-DAAO (10 mU, 50 ng of enzyme) is cytotoxic to various tumor cell lines. In addition, the use of DAAO variant enzyme allowed nanomaterials to show the equal cytotoxicity with an oxygen concentration of 21% and 2.5%. This suggests that the catalytic reaction of the DAAO variant enzyme is no longer restricted by oxygen concentration and can act even in the center of solid tumors.

Fuentes-Baile et al. [10] loaded CLytA-DAAO on magnetic nanoparticles (MNPs) to synthetize antitumor nanomaterials with starvation therapy in collaboration with oxidative therapy. CLytA acts as a junction molecule to help DAAO been loaded on surface-functionalized MNPs in a non-covalent bond manner. For one thing, DAAO is used to oxidative amino acids present in the tumor to achieve tumor-starving effects. For another thing, it generates large amounts of H_2_O_2_ to exert strong cytotoxicity. Interestingly, activation of a classical apoptosis pathway caused the death of glioblastoma, while it exhibited in the form necrosis in colon and pancreatic cancers [83]. In a recent study, this team [84] found that CLytA-DAAO fixed via MNPs increased DAAO stability and prolonged peripheral cycle time, inducing cytotoxicity more efficiently than free CLytA-DAAO.

## Synergistic tumor therapy system of metal-based nanoparticles carrying oxidases

Insufficiency of endogenous H_2_O_2_ in tumor cells results in limited •OH generated by Fenton/Fenton-like reaction, which severely reduces the therapeutic effect of CDT. Without modifications of loading with multiple functional factors, metal-based nanoplatform always fails to induce significant damages to tumor cells. However, new problems show up after modifications: (1) The complexity of materials increases the difficulty of synthesis. (2) The modified functional factors may not be able to effectively synergize. Back to the beginning of the problem, how to increase the content of H_2_O_2_ in the cell has become the key to solve the problem. It is known that that GOx, LOX, and AAO can respectively oxidize and decompose glucose, lactate, and amino acid to produce a large amount of H_2_O_2_, and produce acidic substances to reduce pH. Sufficient H_2_O_2_ is generated and an appropriate pH is formed, which maximizes the antitumor effect of CDT. At the same time, the lack of nutrients in the tumor mediates tumor starvation therapy. The perfect cooperative relationship between the CDT and starvation therapy makes the “metal-oxidase” cascade catalytic system the best option. We only detailly discuss the classical “metal-oxidase” cascade catalytic system due to the space reason.

### Synergistic tumor therapy system of iron and glucose oxidase

**Fig. 6:**
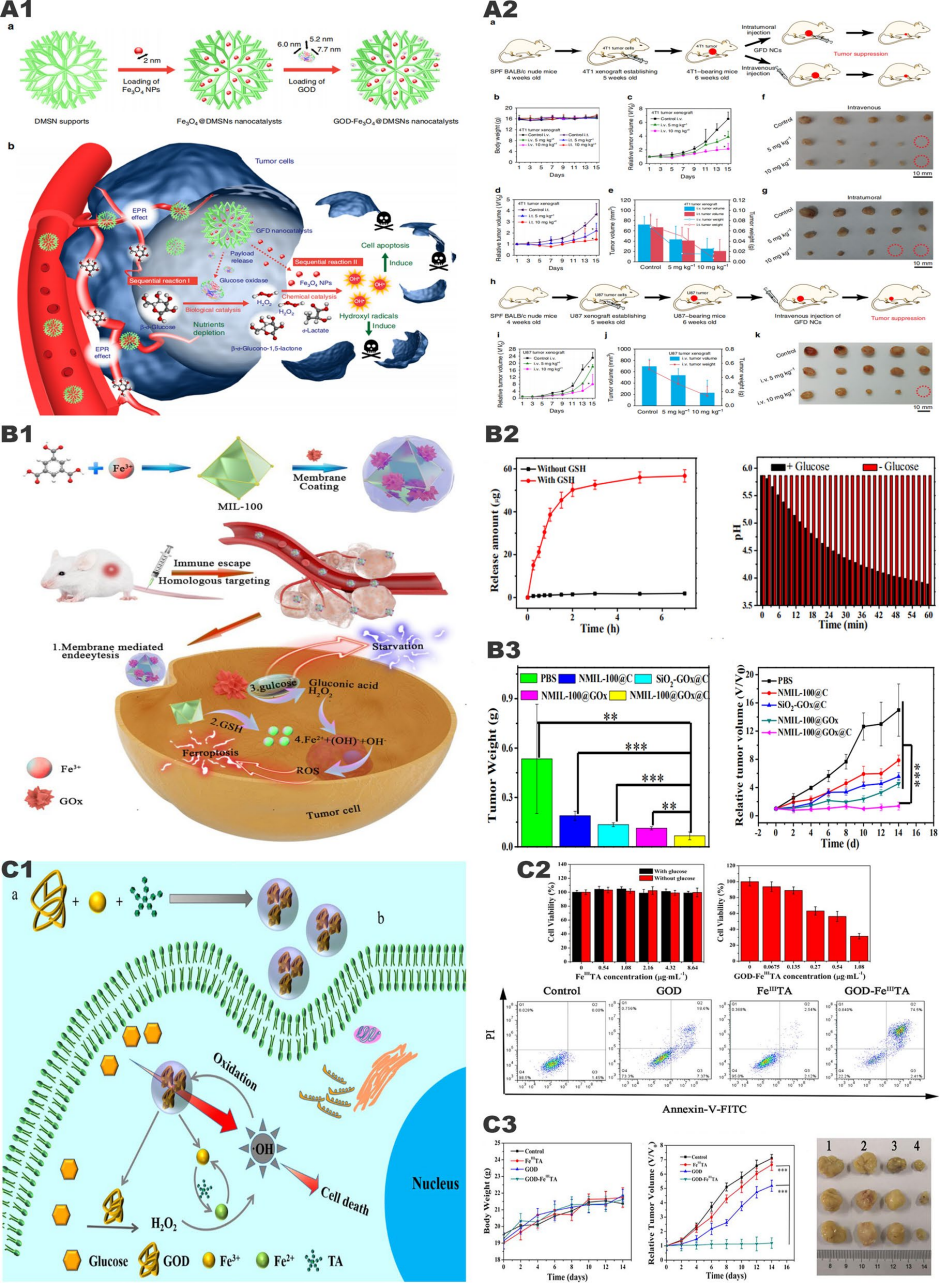
"Iron-GOx" synergistic anti-tumor effect. **A1** Fabrication and catalytic-therapeutic schematics of sequential GFD NCs. **A2** In vivo catalytic-therapeutic performance of GOx-Fe_3_O_4_@DMSN against 4T1 and U87 tumor xenografts. Reproduced with permission from Ref [59], copyright © 2017 Springer Nature. **B1** Schematic illustration of the preparation of NMIL-100@GOx@C and the cascade processes for cancer therapy. **B2** GSH triggered Fe^2+^ release from NMIL-100. And the pH value changes of NMIL-100@GOx@C solution with or without glucose. **B3** Average tumor weights and tumor growth curve in different treatment groups. Reproduced with permission from Ref [86], copyright © 2020, American Chemical Society. **C1** Synthetic procedure and corresponding therapeutic principle of GOx-Fe^III^TA nanocomposites. **C2** Viability of MCF-7 cells after the incubation with different concentrations of Fe^III^TA or GOx-Fe^III^TA nanocomposites. And cell apoptosis of MCF-7 cells after the incubation with different agents. **C3** Average body weight and relative tumor volume in different groups subjected to various treatments. Reproduced with permission from Ref [88], copyright © 2019 IOP Publishing

Huo et al. [59] prepared mesoporous silica loaded with GOx and Fe_3_O_4_ nanoparticles GOx-Fe_3_O_4_@DMSN (Fig. 6, A1). In this system, GOx catalyzes the oxidation and decomposition of glucose to produce a large amount of H_2_O_2_ and generates numerous •OH through the Fenton reaction catalyzed by Fe^2+^, which further induces tumor cell apoptosis (Fig. 6, A2, A3). However, the catalytic system has poor targeting ability, and the catalytic reaction only occurs on the surface of Fe_3_O_4_ nanoparticles, resulting in low catalytic efficiency. Interestingly, Ranji-Burachaloo et al. [85] used pH-sensitive zeolitic imidazolate frameworks 8 (ZIF-8) with GOx and hemoglobin to improve the defects mentioned above. ZIF-8 is stable under the neutral pH conditions of normal tissues but rapidly decomposes in the acidic TME, releasing GOx and consuming glucose to generate H_2_O_2_. Then a large amount of H_2_O_2_ acts on hemoglobin to trigger the release of Fe^2+^, which further catalyzes the Fenton reaction. In vivo and in vitro experiments have confirmed that the nanoparticles have good tumor inhibitory effects and biological safety. Application of ZIF-8 makes nanomedicine pH-sensitive, which greatly improves its targeting function and biosafety. In addition, we can also cover the nanoparticles with tumor cell membranes (Fig. 6, B1). Cancer cell membranes will give nanoparticles the ability to homologous targeting and immune escape, which is conducive to the effective accumulation of nanomedicine at tumor sites and maximize the antitumor effect (Fig. 6, B2, B3) [86].

In the general “iron-oxidase” cascade catalytic system, Fe^2+^ is oxidized to Fe^3+^ with lower catalytic ability via the Fenton reaction, resulting in a great decrease in the efficiency of the Fenton reaction. This means that a large amount of Fe^2+^ nanoparticles is needed to generate enough active oxygen, but increasing the dose will also cause unnecessary damage to normal tissues. In response to this problem, Zhang et al. [87] proposed adding TA to the system of iron ions and oxidases that catalyze the reaction. TA can reduce Fe^3+^ to Fe^2+^ to enhance the Fenton reaction. Du et al. [88] also adopted a similar idea, constructing Fe^3+^-tannic acid (Fe^III^TA) nanocarriers and loading GOx (Fig. 6, C1). Compared with traditional Fe^2+^/Fe^3+^, Fe^III^TA nanocomposites exhibit higher catalytic activity in the conversion of H_2_O_2_ to highly toxic •OH due to the Fe^3+^ reduction effect mediated by TA, which significantly improves the efficiency of the Fenton reaction. In vitro experiments show that the •OH prepared by GOX-Fe^III^TA nanocomposites can not only obtain good antitumor effects at low concentrations but also promote the degradation of nanocomposites. When the concentration was only 1.08 ug/ml, the tumor cell apoptosis rate reached 76.91% (Fig. 6, C2). In vitro experiments showed that Fe^III^TA can well inhibit primary tumors (Fig. 6, C3).

### Synergistic tumor therapy system of copper and glucose oxidase

At present, many experiments have confirmed the antitumor effect of the “iron-GOx” synergistic catalytic system, but this system still has the following shortcomings: Fe^2+^ catalyzes the Fenton reaction at a lower rate, and Fe^3+^ is converted to Fe^2+^ at a slow rate and highly depend on the acidic environment. Therefore, researchers loaded reducing substances (TA, H_2_S, etc.) on the “iron-GOx” system for Fe^3+^/Fe^2+^ conversion to increase the rate of Fenton reaction. However, this will make the synthetize of nanoparticles more complicated and limit the stability and practicality of the nanoparticles. We found that on the one hand, Cu^2+^ can react with GSH to generate Cu^+^ and GSSG by redox reaction, which not only consumes reducing GSH, but also newly generates Cu^+^ to enhance the catalytic efficiency of Fenton-like reaction. On the other hand, we noticed that compared with Fe^2+^/Fe^3+^, Cu^+^/Cu^2+^ has a higher catalytic rate of Fenton-like reaction, a faster conversion rate, and a wider pH range. Therefore, the “copper-GOx” system has more advantages than the “iron-GOx” system.

**Fig. 7:**
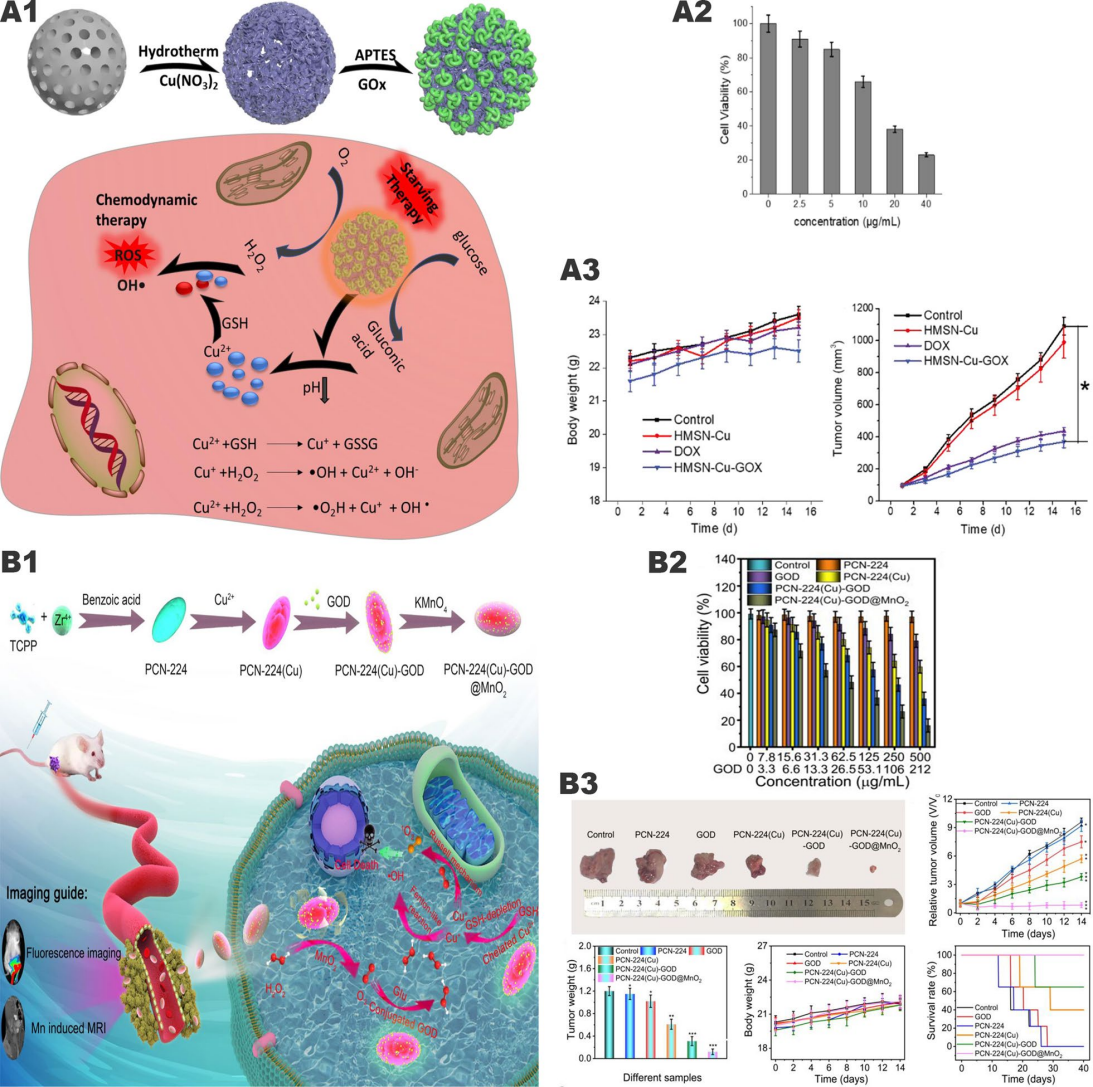
Synergistic effect of "copper-GOx" cascade catalytic system in tumor therapy. **A1** Scheme of synthetic process and therapeutic mechanism of HMSN-Cu-GOD. **A2** Cytotoxicity of HMSN-Cu-GOD treatment for MCF-7 cells after 24 h of incubation. **A3** Body weight and tumor volumes change during 15 days of observation periods in different groups subjected to various treatments. Reproduced with permission from Ref [89], copyright © 2020 Elsevier. **B1** Schematic illustration of the main synthesis procedures and antitumor mechanism of PCN-224(Cu)-GOx@MnO_2_ nMOFs. **B2** Cell viability with different treatment. **B3** In vivo anticancer efficacy of PCN-224(Cu)-GOx@MnO_2_ nMOFs. Reproduced with permission from Ref [90], copyright © 2020, American Chemical Society

Wang et al. [89] mounted Cu^2+^/Cu^+^ on mesoporous silica and modified GOx on the surface to construct a pH-sensitive cascaded nanocatalyst HMSN-Cu-GOx (Fig. 7 , A1). Through the EPR effect, HMSN-Cu-GOx accumulates in the tumor area. Then GOx catalyzes the conversion of glucose into H_2_O_2_ while lowering the pH value, thereby degrading the pH-sensitive HMSN-Cu, thereby releasing Cu^2+^/Cu^+^ and inducing CDT. In vitro experiments showed that when the glucose concentration was 100 ug/mL, the survival rate of MCF-7 cells treated with 40 ug/mL HMSNCu-GOx was only 23% (Fig. 7, A2). In the in vivo experiment, MCF-7 tumor-bearing mice were divided into 4 groups: (1) PBS control group, (2) HMSN-Cu (10 mg/kg), (3) DOX group (5 mg/kg), (4) HMSN-Cu-GOx (10 mg/kg). When the tumor volume reached about 100 mm^3^, the above drugs were injected intravenously every 2 days. After 15 days, the tumor suppression rates in the DOX and HMSN-Cu-GOx treatment groups were 65% and 79%, respectively (Fig. 7, A3). However, the HMSN-Cu group had no therapeutic effect. It can be seen that the “copper-GOx” cascade catalytic system has better antitumor effects than chemotherapeutic drugs alone.

However, the GOx of the above-mentioned cascade nanocatalyst is located on the outside, which will increase the probability of damage to normal cells. Wang et al. [90] designed a nanoparticle PCN-224(Cu)-GOx@MnO_2_ based on the “copper-GOx” system (Fig. 7, B1). The particles are wrapped by MnO_2_ to reduce the possibility of GOx contacting normal tissues. At the same time, MnO_2_ catalyzes H_2_O_2_ in the TME to generate oxygen, which increases the catalytic efficiency of GOx. In vivo experiments have verified that PCN-224(Cu)-GOx@MnO_2_ has little effect on normal tissues and has a good tumor inhibitory effect (Fig. 7, B2, B3).

### Synergistic tumor therapy system of iron and lactate oxidase

**Fig. 8:**
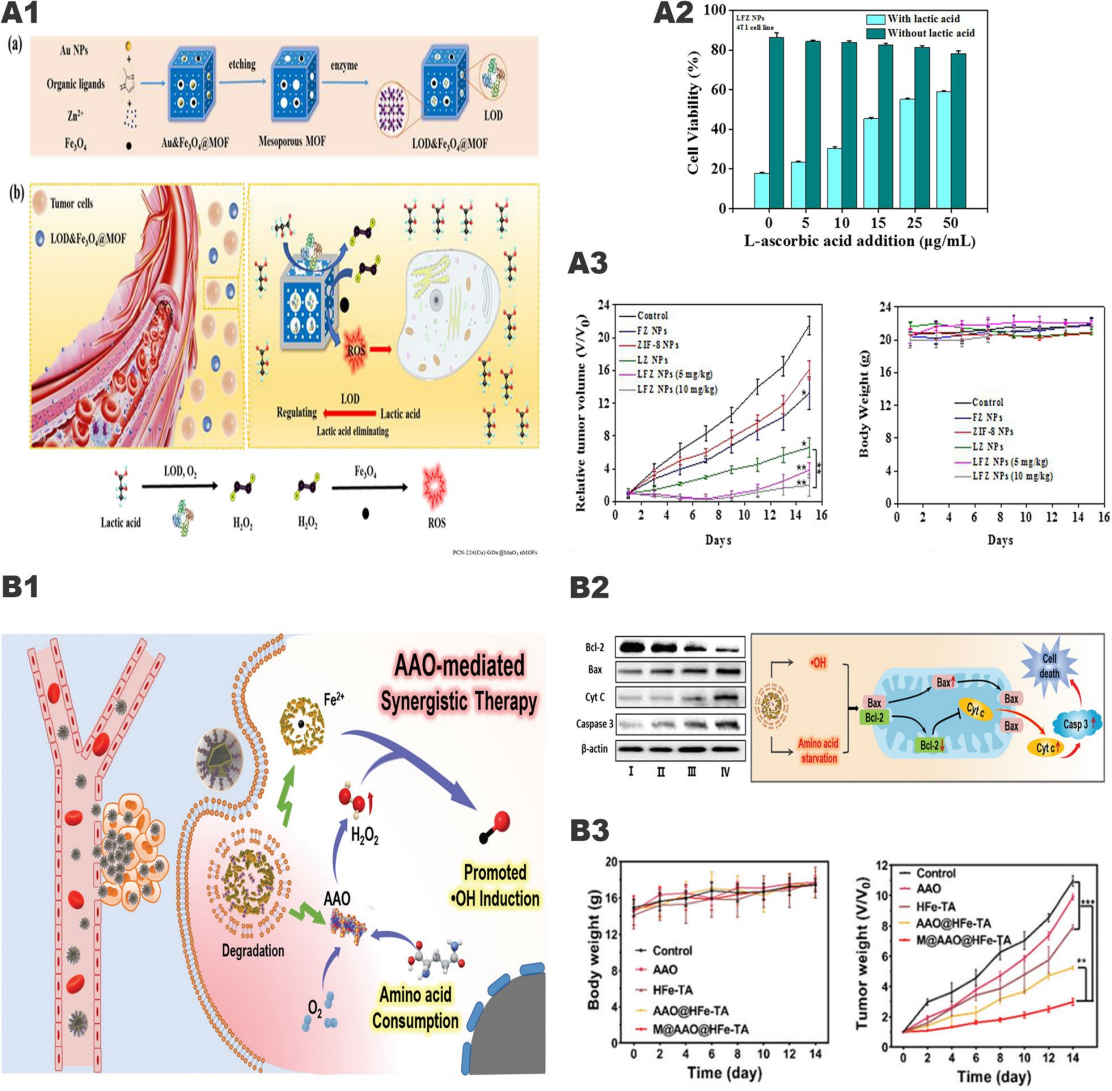
The synergistic anti-tumor effect of " iron-LOX " and "iron-AAO". **A1** The tandem biological–chemical catalytic reactions for effective catalytic tumor treatment based on the characteristic of TME. **A2** Cell rescue profiles of 4T1 cells’ cytotoxicity induced by LFZ NPs (10 μg/mL) with different concentrations of l-ascorbic acid. **A3** The relative tumor volume and the body weight under different treatments. Reproduced with permission from Ref [91], copyright © 2020, American Chemical Society. **B1** Schematic illustration for the functioning mechanism of M@AAO@HFe–TA nanocapsules. **B2** Protein expression of Bcl-2/Bax/Cyt C/caspase 3 mitochondrial apoptotic pathway in 4T1 cells under different treatments. And schematic illustration of in vitro antitumor mechanism of M@AAO@HFe–TA nanocapsules. **B3** Body weight and tumor volume in different mice groups during the 14 days of treatment. Reproduced with permission from Ref [94], copyright © 2020, Royal Society of Chemistry

Increasing evidences have shown that the accumulation of lactate in the TME changes the living environment and immune status of the tumor. Excessive lactate can not only be considered as nutrient but also involved in the proliferation, metastasis, and recurrence of the tumor. However, regarding lactate as the target in tumor therapy has not received widespread attention. The “iron-LOX” cascade catalytic system may be one of the important breakthroughs for tumor treatment. In this system, LOX can consume lactate inside and outside the tumor cells, changing the survival state of the tumor and generating excessive H_2_O_2_. Subsequently, the generated H_2_O_2_ can be converted into strong oxidizing •OH through Fenton reaction, thereby inducing tumor cell apoptosis. Zhou et al. [91] prepared layered porous ZIF-8 nanovectors based on the above principles and simultaneously loaded with LOX and Fe_3_O_4_ nanoparticles, known as LFZ NPs (Fig. 8, A1). In vivo and in vitro experiments have confirmed that LFZ NPs have good antitumor ability and reliable biosafety (Fig. 8, A2, A3). This system has similarities with the above-mentioned “iron-GOx” system, but most of the current related researches are just at the preliminary stage and need further enhancements.

### Synergistic tumor therapy system of iron and amino acid oxidase

Amino acids are the basic components of tumor cell proteins. The consumption of amino acids is an effective way to destroy the tumor cytoskeleton and the metabolic balance in tumor cells [92, 93]. Based on this principle, Chu et al. [94] used hollow Fe^3+^/TA nanocapsules (HFe-TA) loaded with AAO and wrapped it with cancer cell membrane to construct a nanocapsule (M@AAO@HFe-TA) for the first time, for the treatment of tumors (Fig. 8, B1). In this system, the intracellular transport of AAO molecules can effectively catalyze the oxidative deamination reaction, consuming a large number of amino acids. At the same time, the up-regulation of intracellular acid and H_2_O_2_ concentrations further promoted the Fenton reaction mediated by HFe-TA and enhanced the production of cytotoxic •OH. The 4T1 cancer cell membrane on the surface of the nanocapsule plays a protective role in preventing AAO exposure and potential cytotoxicity. At the same time, using of cancer cell membranes to modify the surface of the nanoparticles can empower it the ability to target tumor cells and avoid immune clearance. Both in vitro and in vivo studies have elucidated that M@AAO@HFe-TA can eliminate tumors through the Bcl-2/Bax/cytc/caspase3 mitochondrial apoptosis pathway (Fig. 8, B2), and has a good ability in triggering the redox of intracellular amino acid and catalyzing the Fenton reaction (Fig. 8, B3).

## Comprehensive antitumor therapeutic system of “metal-oxidase” system combined with other therapies

### “Metal-oxidase” system in combination with chemotherapy

The toxic side effects on normal cells and severe drug resistance are main restrictions of the application of chemotherapy. Studies have shown that the TME is an “euthanole” for tumor cells, considering it as an important factor of drug resistance to various chemotherapeutic agents [95]. In the few decades, large amounts of nanoparticles have been applied as vectors to deliver chemotherapeutic drugs with the development of Nanodrug transport systems [96]. Many of these transition-state metal-based nanomaterials can not only serve as a catalyst for the Fenton/Fenton-like reaction, but also as excellent drug vectors to load chemotherapeutic agents. There are several advantages of using nanovectors to deliver chemotherapeutic agents: (1) Drugs can be accumulated selectively in tumors, successfully enhancing the specific killing ability of tumors [97]. (2) Chemotherapeutic agents are all protected by nanovectors before reaching tumor tissues, greatly minimizing systemic toxic adverse effects [98]. (3) There are smaller needs of chemotherapeutic drugs due to the antitumor effects nanomaterials own [99]. (4) It is possible to exert better efficacy because of the prolonged cycle time of the chemotherapeutic agents [100]. (5) Nanomaterials can remold the TME, overcoming drug resistance to chemotherapeutic agents [101, 102]. The “metal-oxidase” system mentioned above has strong antitumor ability on its own and possess good biosafety at the same time. If been well-designed, the “metal-oxidase” system can be applied as an excellent chemotherapeutic drug carrier, which has at least five advantages listed above. Therefore, the combination of the “metal-oxidase” system with chemotherapeutic agents would be greater and safer.

**Fig. 9:**
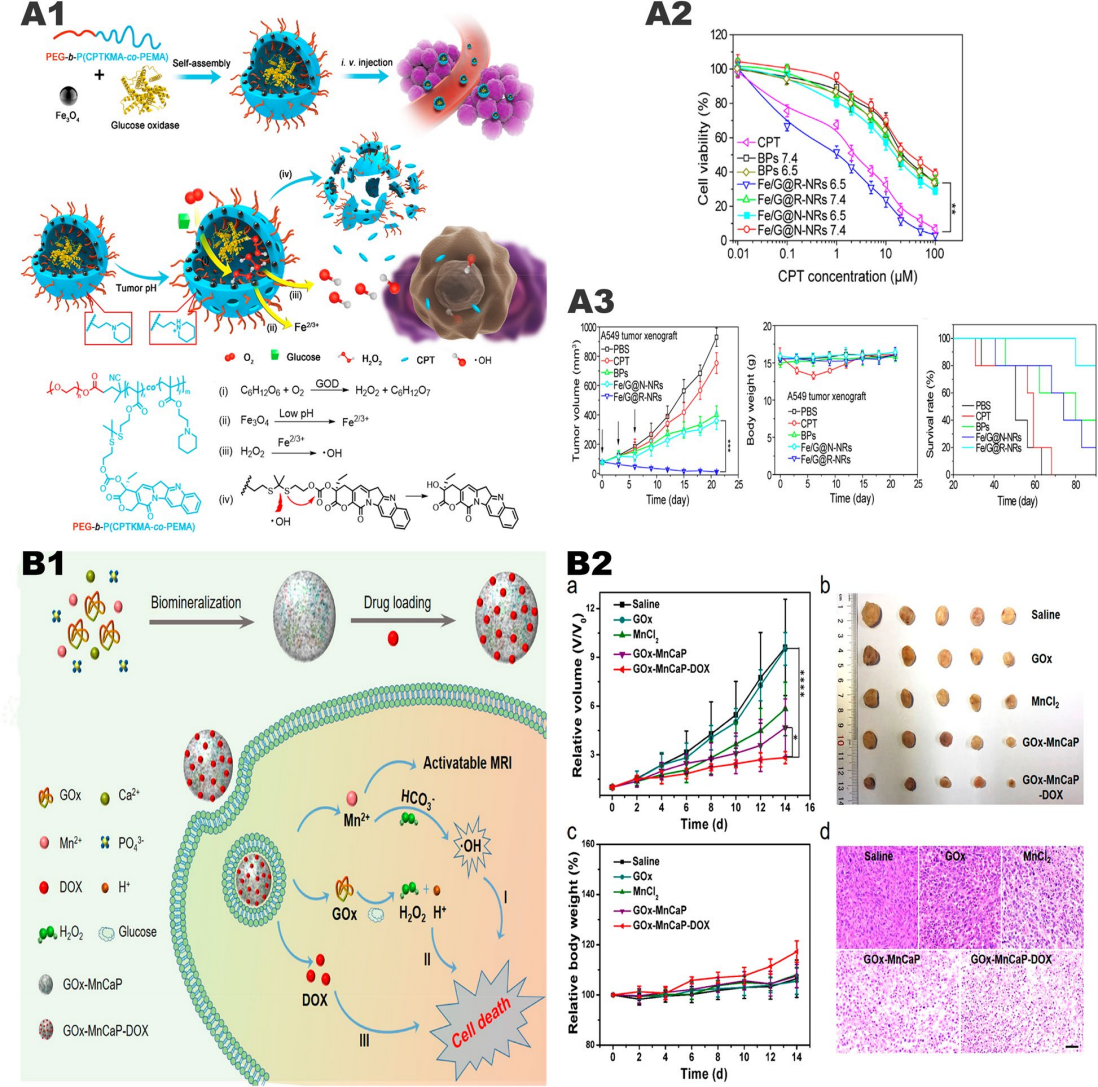
The synergistic anti-tumor system of "metal-oxidase" system combined with chemotherapy. **A1** Diagram of how Fe/G@R-NRs is synthesized and how it is in an acidic environment at disassembly further and induces multiple antitumor responses. **A2** Cytotoxicity against A549 cells at various CPT-equivalent concentrations. **A3** Time-dependent A549 tumor growth profiles. The arrows indicate the injection time. Mice body weight change over the time during the treatment. The survival curve of A549 tumor-bearing mice after various treatments. Reproduced with permission from Ref [103], copyright © 2019, American Chemical Society. **(B)** **B1** schematic illustration of GOx-MnCaP-DOX applied for MRI-monitored cooperative cancer therapy. **B2** Average body weight and relative tumor volume in different groups subjected to various treatments. And H&E staining images of tumor tissues excised from the mice at day 14. Reproduced with permission from Ref [105], copyright © 2019, American Chemical Society

Ke et al. [103] prepared a pH-sensitive polymer nanoreactor Fe/G@R-NRs (Fig. 9, A1) with a pH-sensitive permeable membrane on the periphery and containing Fe_3_O_4_, GOx, and camptothecin (CPT). In the acidic TME, Fe/G@R-NRs release GOx and Fe^2+^, inducing a large amount of •OH. Since CPT and the nanoreactor are connected by thioketal bond, When large amounts of •OH are present, the thioketal bond breaks and releases CPT [104]. In this process, synergistic tumor treatment including starvation therapy, CDT, and chemotherapy was realized. In the in vitro experiment, it was found that the efficiency of •OH production and release of CPT achieved to the highest at pH 6.5, while there was only a very small amount of •OH production and CPT release when the pH was 7.4 (Fig. 9, A2). In vivo experiments found that the tumor was completely suppressed after 21 days of Fe/G@R-NRs treatment, and the weights of the mice did not change (Fig. 9, A3). In addition, in vivo experiments also compared Fe/G@R-NRs with using CPT alone, and the results showed that Fe/G@R-NRs had more obvious antitumor effects and better prognosis.

Fu et al. [105] used an in-situ biomimetic mineralization method to mineralize GOx with manganese-doped calcium phosphate (MnCaP) and loaded DOX to form spherical nanoparticles GOx-MnCaP-DOX (Fig. 9, B1). The GOx-driven oxidation reaction can effectively remove glucose in the tumor for starvation therapy, and then the excessive H_2_O_2_ is converted into highly toxic •OH for CDT through a Fenton-like reaction mediated by Mn^2+^. GOx-MnCaP-DOX can combine starvation therapy, Mn^2+^-mediated CDT, and DOX-induced chemotherapy. In vivo and in vitro experiments have shown that its therapeutic effect is significantly better than single-agent therapy (Fig. 9, B2).

### “Metal-oxidase” system in combination with hypoxia-activated drugs

Tumor hypoxia is also an attractive treatment target because it is one of the main differences between tumors and normal tissues. Hypoxia-activated drugs selectively show high toxicity in hypoxic cells while only show low toxicity in areas of higher oxygen tension such as normal tissues. Therefore, hypoxia-activated drugs have potential in specifically killing hypoxic cells, thereby turning tumor hypoxia from a defect into an advantage for selective treatment. Adopting of oxidases largely aggravates the hypoxia degree of the TME and increases the antitumor effect of hypoxia-activated drugs.

**Fig. 10:**
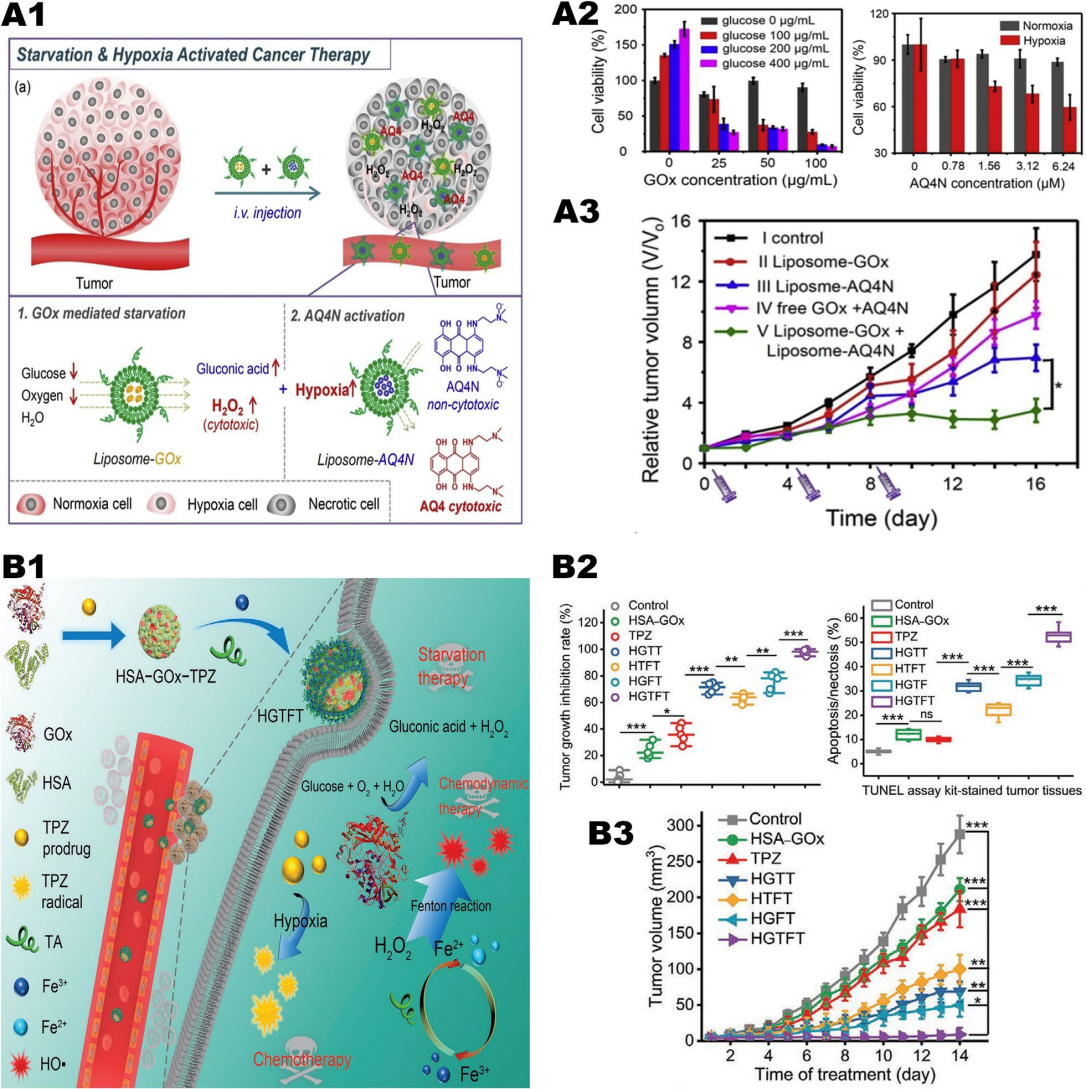
"Metal-oxidase" system combined with hypoxia activation therapy synergistic anti-tumor system. **A1** Scheme illustrating the design of combining starvation and hypoxia-activated therapy by co-delivery of liposome-GOx and liposome-AQ4N into tumors. **A2** Relative viabilities of 4T1 cells after 24 h incubated with different concentrations of glucose and liposome-GOx. And relative viabilities of 4T1 cells after 48 h incubation with different concentrations of liposome-AQ4N under the normoxia or hypoxia culture condition. **A3** Tumor growth curves of mice after various different treatments indicated. Reproduced with permission from Ref [48], copyright © 2018 Elsevier. **B1** Schematic diagram showing the fabrication of HGTFT nanoreactors and their applications for starvation therapy, CDT, and chemotherapy. **B2** Tumor growth inhibition rates of different formulations. And apoptosis/necrosis rates determined by the TUNEL assay. **B3** Tumor growth profiles of the mice in the different groups. Reproduced with permission from Ref [107], copyright © 2020 WILEY–VCH

Researchers used liposome nanovectors to combine GOx-based starvation therapy with AQ4N-based hypoxia activation therapy, and innovatively developed a novel tumor treatment strategy (Fig. 10, A1) [48]. The GOx and AQ4N were encapsulated with polyethylene glycol liposomes. After intravenous injection, due to the long-term blood circulation of liposomes, highly effective tumor homing can be observed. In vivo experiments showed that GOx can specifically block the glucose supply of tumor cells, decrease the oxygen in the tumor, and produce cytotoxic H_2_O_2_ molecules to kill tumor cells (Fig. 10, A3). AQ4N is reduced to hydrophobic AQ4N in a hypoxic environment, and then be released from liposomes. As a DNA-related insert or topoisomerase II toxic substance, AQ4N can diffuse into the cytoplasm through the lysosomal membrane, and then enter the nucleus and combine with nuclear DNA. This process eventually leads to tumor cell death (Fig. 10, A2).

TPZ is a representative hypoxia-activated drug [106]. Under hypoxia, TPZ shows 300 times more toxic effects on tumor cells than under aerobic conditions. As a prodrug, TPZ can be stimulated and activated by a single-electron reduction reaction under hypoxic conditions, resulting in cytotoxicity. Although the tumor area is a hypoxic environment, it is still not enough to activate the effect of TPZ. How to decrease the oxygen concentration in the tumor area and improve the therapeutic effect of TPZ is a current problem confusing us. We propose a treatment plan in which part of the oxygen can be consumed during therapy, thereby improving the treatment effect of TPZ. Guo et al. [107] prepared TPZ-human serum albumin (HSA)-GOx mixture and modified it with Fe^3+^ and TA to obtain a self-amplifying nanoreactor named HSA-GOx-TPZ-Fe-TA (HGTFT) for continuous and cascade cancer treatment (Fig. 10, B1). The HGTFT nanoreactor has good biocompatibility and in vivo stability, thus can be targeted to accumulate in the tumor area through the EPR effect. Among them, TA promotes the conversion of Fe^3+^/Fe^2+^. The HGTFT nanoreactor can effectively consume glucose and oxygen for starvation treatment while generating •OH to induce CDT and provide a hypoxic environment for TPZ-mediated chemotherapy. Both in vivo and in vitro experiments have shown that HGTFT has good antitumor effects and reliable biosafety (Fig. 10, B2, B3). Compared with using TPZ alone for antitumor therapy, HGTFT has shown obvious advantages.

### “Metal-oxidase” system in combination with photothermal therapy

PTT is a photothermal agent with good light-to-heat conversion performance, which can finally reach the tumor site through local injection or tail vein injection. After the photothermal agent reaching the tumor site, it can generate massive heat under the irradiation of near-infrared light, which will increase the temperature of the tumor tissue (often quickly above 42 °C) and cause further tumor cell death [108]. Studies have shown that when the temperature is increased from 20 °C to 50 °C, the rate of the Fenton reaction is increased by 4 times, which greatly promotes the efficacy of CDT [108]. The exciting news is that most of the metal-based nanoparticles that catalyze the Fenton reaction are good photothermal agents. Therefore, we can further improve the efficiency of the “metal-oxidase” system by integrating PTT and maximize the synergy of CDT/starvation therapy/PTT.

**Fig. 11:**
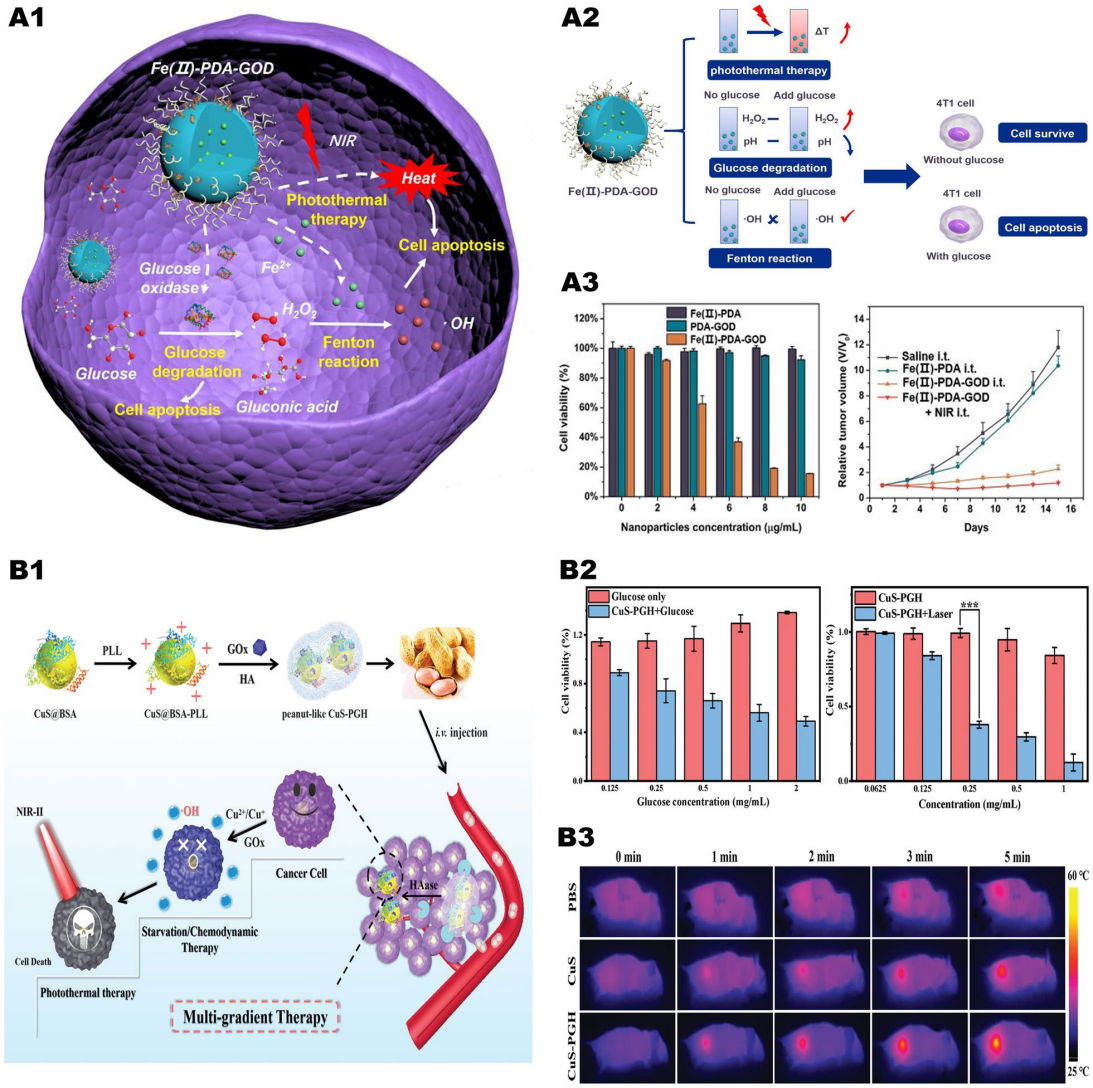
Synergistic anti-tumor system of "metal-oxidase" system combined with photothermal therapy. **A1** Schematic illustration of TME-based Fe(II)-PDA-GOD nanosystems for efficient cancer therapy by combining glucose degradation, Fenton reaction and photothermal therapy. **A2** Schematic illustration of photothermal effect and catalytic performance of Fe(II) -PDA-GOD NPs and their in vitro antitumor effect. **A3** The antitumor effects of nanoparticles in vivo and in vitro under different treatments. Reproduced with permission from Ref [109], copyright © 2019, American Chemical Society. **B1** Schematic illustration of the construction of CuS-PGH NMs for multi-gradient antitumor therapy. **B2** Changes of 4T1 cell viability after different treatments. **B3** The real-time thermal images recorded using an IR thermal camera. Reproduced with permission from Ref [110], copyright © 2020, Royal Society of Chemistry

Zhu et al. [109] prepared a TME-reactive nanosystem Fe(II)-PDA-GOx (Fig. 11, A1, A2) based on polydopamine (PDA). GOx and Fe^2+^ form an “iron-GOx” cascade catalytic system, which induces the synergy of starvation therapy and CDT. When irradiated with NIR, due to the photothermal effect of PDA, a large amount of heat is generated at the tumor site, which increases the speed of each step of the cascade catalytic reaction. In the in vivo test, Fe(II) -PDA alone has no antitumor effect, while Fe(II) -PDA-GOx has a strong antitumor effect, which produces the excellent antitumor effect when NIR is present (Fig. 11, A3). This showed that the antitumor effects of CDT induced by the Fenton reaction catalyzed by metal ions fail to meet our demands, but it can be solved by combining it with oxidases to form a synergistic catalytic system.

Similarly, we can make use of the photothermal effect of copper-based nanoparticles to improve the catalytic efficiency of the “copper-GOx” system. Chen et al. [110] used poly (L-lysine) (PLL)/GOx-HA shell as a vector and then loaded CuS-PGH nanoparticles (Fig. 11, B1). CuS-PGH has a strong NIR-II absorption capacity, can achieve effective photothermal ablation of tumor cells, and greatly enhance the efficacy of Cu^2+^/Cu^+^-mediated CDT (Fig. 11, B3). This multi-gradient treatment strategy has considerable antitumor effects and minimal non-specific damage (Fig. 11, B2), providing a novel way for precise tumor treatment.

### “Metal-oxidase” system in combination with immunotherapy

In recent years, tumor immunotherapie aimed at activating the patients’ innate immune system have shown great promise. It has now been established that the tumor cell death in the primary site can release TAAs [111]. DCs are able to capture these antigens, and then present these antigens to the T cell receptor via a major histocompatibility complex (MHC) after migrating to immune organs such as spleen or lymph nodes. Ultimately, T cell-mediated long-term tumor immune are successfully triggered [112].

However, there are many deficiencies of using immune checkpoint inhibitors alone: (1) Treatment effects are limited due to the immune-suppressed TME. (2) A large demand of immune checkpoint inhibitors results in high cost of treatment. (3) The large-dose and systemic drugs are prone to induce dose-dependent autoimmune diseases. Thus, finding out alternatives to immune checkpoint inhibitors or combing them with immune agonists targeting tumors is crucial to solve the problem. The “metal-oxidase” system combined with immunotherapy is exactly our ideal solution, which has the following advantages: (1) Immune checkpoint inhibitors loaded with nanoparticles can specifically target tumor tissues to avoid additional adverse effects. (2) Nanomaterials directly kill tumor cells and then release TAAs, further activating tumor immunity. (3) The combined therapy can reverse the immunosuppressive state of the TME and enhance the efficacy of immune checkpoint inhibitors, such as avoiding lactate accumulation, reducing acidity, and alleviating hypoxia. (4) Tumor immune activation caused by the “metal-oxidase” system greatly decreases the demand of immune checkpoint inhibitors and achieves the same therapeutic effect, reducing the adverse effects and also lightening the burden of patients. Therefore, the combination of tumor immunotherapy with the “metal-oxidase” system is pivotal for the treatment of primary, recurrent or metastatic tumors [113, 114].

#### Immunoactivation effects of metal-based nanomaterials

Transition-state metal-based nanomaterials can catalyze the Fenton/Fenton-like reaction, generating a large number of strongly oxidative •OH used to kill tumor cells. The death of tumor cell is accompanied by the release of TAAs, which further induces DCs maturation to initiate innate and adaptive immunity. Furthermore, it has been shown that Mn^2+^ is a second cGAS activator besides dsDNA and acts in the activation of the cGAS-STING pathway, further activating tumor immunity via mediating DCs maturation [41, 115].

**Fig. 12:**
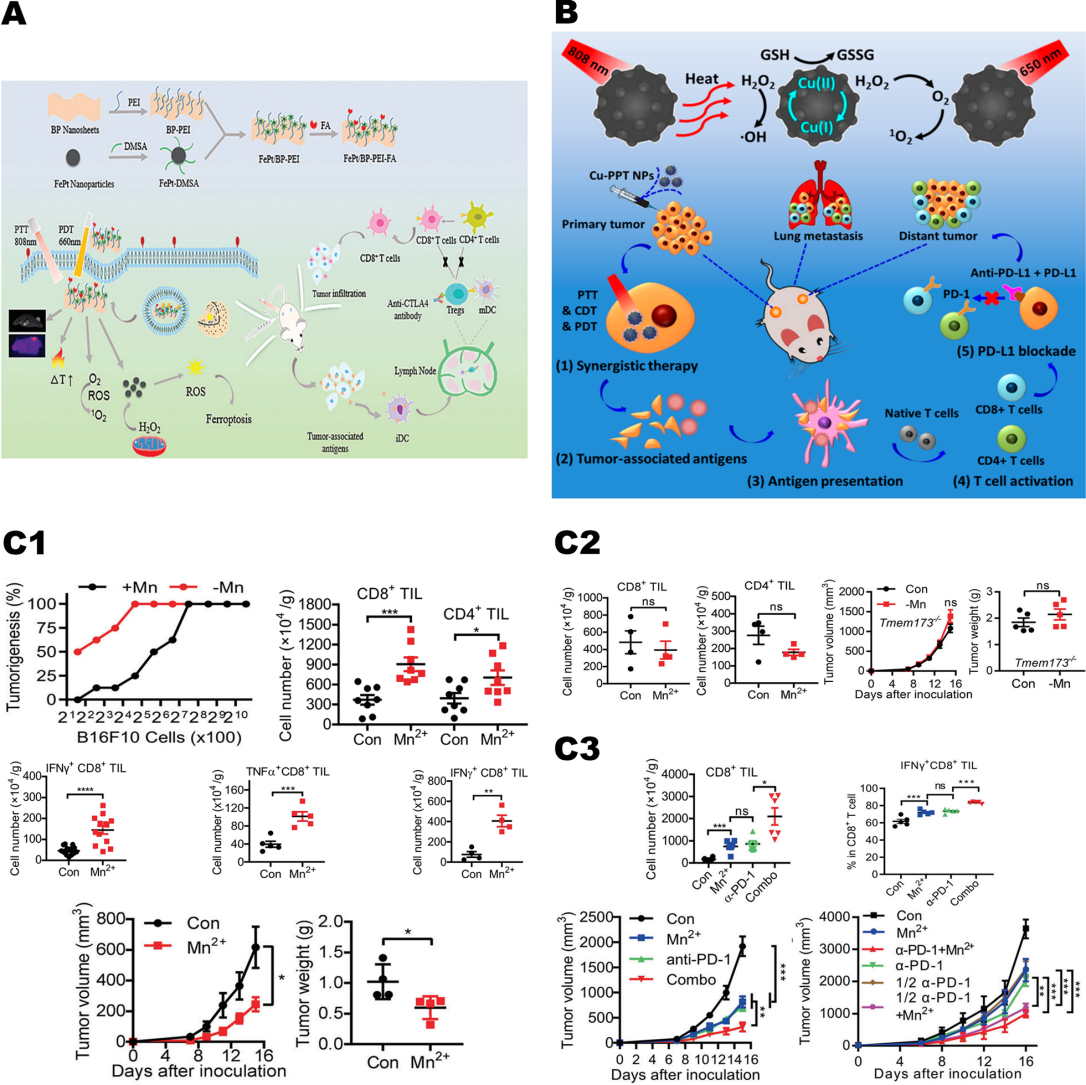
Metal-based nanoparticles mediate immune activation. **A** Schematic illustration of the FePt/BP–PEI–FA NCs enhanced immunotherapy. Reproduced with permission from Ref [16], copyright © 2020, Royal Society of Chemistry. **B** Antitumor effect of Cu-PPT nanoparticles combined with immune checkpoint inhibitors. Reproduced with permission from Ref [116], copyright © 2020, American Chemical Society. **C1** Mn is essential for immune responses against tumors. Mn^2+^ stimulates CD8^+^ T cells activation and promotes DCs maturation and antigen presentation. **C2** Mn^2+^ mediated cGAS-STING pathway to activate tumor immunity. **C3** Mn^2+^ boosts antitumor immunotherapy in mice. Reproduced with permission from Ref [41], copyright Copyright © 2020, Springer Nature

Yao et al. [16] synthesized FePt/BP-PEI-FA nanoplatforms by loading FePt nanoparticles on ultrathin black phosphorus nanosheets (BPNs) via polyethylenimine (PEI) (Fig. 12, A). This nanoplatform can induce tumor cell death and release TAAs to further promote tumor immune activation under 808 nm laser by generating large amounts of •OH via Fenton response. In vivo experiments indicated that after treatment with FePt/BP-PEI-FA, combined with the anticytotoxic T lymphocyte-associated protein 4 (anti-CTLA4) checkpoint blockade, tumor immunity was greatly activated and led to complete inhibition of primary and metastatic tumors. Similarly, Hu et al. [116] synthesized Cu-PPT nanoparticles. Under 808 nm laser irradiation, Cu-PPT produced a great quantity of heat and released Cu^+^/Cu^2+^, further consuming GSH and producing large amounts of •OH (Fig. 12, B). This multifunctional nanosystem with a cascade reaction is able to effectively inhibit tumor growth and activate the immune response. Checkpoint blocking therapy by intravenous anti-programmed death ligand 1 (anti-PD-L1) successfully inhibited the growth and metastasis of distant tumors.

It has been reported that Mn^2+^ plays an important role in cGAS-STING pathway activation [117]. Zhao et al. found that Mn^2+^ was a second cGAS activator other than dsDNA and catalyzed an unusual cGAMP synthesis process [115]. cGAS would catalyze the generation of cyclic bird adenytides (cGAMP) when it detects dsDNA that should not have appeared in the cytoplasmic. Subsequently, dimerized STING binds to cGAMP and recruits TBK1 protein, enabling phosphorylation of the interferon regulator (IRF3). Next, IRF3 entering the nucleus induces the generation of type I interferon, which in turn activates innate immunity. Meanwhile, type I interferon can promote the cross-presentation of DCs and initiate tumor-specific CD8-positive T cells [118–121].

Lv et al. [41] discovered that exogenous addition of Mn^2+^ effectively activated the cGAS-STING pathway in human or mouse cells (Fig. 12, C2). On the one hand, it significantly promotes the ability of DCs to present tumor antigens while promote the infiltration of cytotoxic T lymphocytes (CTLs) within tumor tissues and enhancing its specific killing towards tumor cells (Fig. 12, C1). On the other hand, Mn^2+^ can also significantly promote Natural killer cells (NKs) to eliminate tumor cells and enhance immune surveillance of the host. Intratumoral injection of Mn^2+^ completely eliminated the tumors in the vast majority of tumor-bearing mice. All these results suggest that Mn^2+^ is an excellent tumor immune activator that enhances the immune surveillance and immune clearance of tumor cells. Moreover, the combination of Mn^2+^ and PD-1 antibodies in multiple tumor models significantly enhanced the efficacy of PD-1 antibodies while reduced the dose of PD-1 antibodies (Fig. 12), C3). If assuming immunotherapy as a car, then the use of the PD-1 antibody is like removing the brakes of it (loosening the brakes), while the Mn^2+^ is like refueling the engine (on the accelerator). Only then the car can run at its full speed. The above results suggest that the synergy of Mn^2+^ and immunotherapy has great clinical transformation prospects.

#### Immunoactivation effects of oxidases

##### Glucose oxidase and immunotherapy

Wang et al. [17] synthesized Cu-doped cobalt oxide and porous carbon nanocomposites from bilayer ZIF-8@ZIF67 and further loaded them with GOx to get the CuCo(O)/GOx@PCNs hybrid nanozyme. CuCo(O) can react with H_2_O_2_ to generate oxygen, relieving tumor hypoxia, restoring GOx enzymatic activity, further enhance starvation therapy. Porous nanocarbons have a photothermal conversion efficiency of up to 40.04%, enabling not only thermally ablate tumors, but also accelerate the generation of H_2_O_2_ catalyzed by GOx. Under the strong oxidative and photothermal effects of H_2_O_2_, tumor cells were killed and then released TAAs to activate tumor immunity. Treatment with CuCo(O)/GOx@PCNs significantly increased CD8^+^ T cells and CD4^+^ T cells in primary tumor tissues, suggesting decreased regulatory T cells (Treg cells). Meanwhile, the levels of cytokines (TNF-α, IFN-γ and IL-12) secreted by DCs were significantly upregulated. Above all, similar immune activation effects were observed in the tumor metastasis site after the primary site being treated with CuCo(O)/ PCNs/GOx, which resulted in complete suppression of tumor metastasis.

**Fig. 13:**
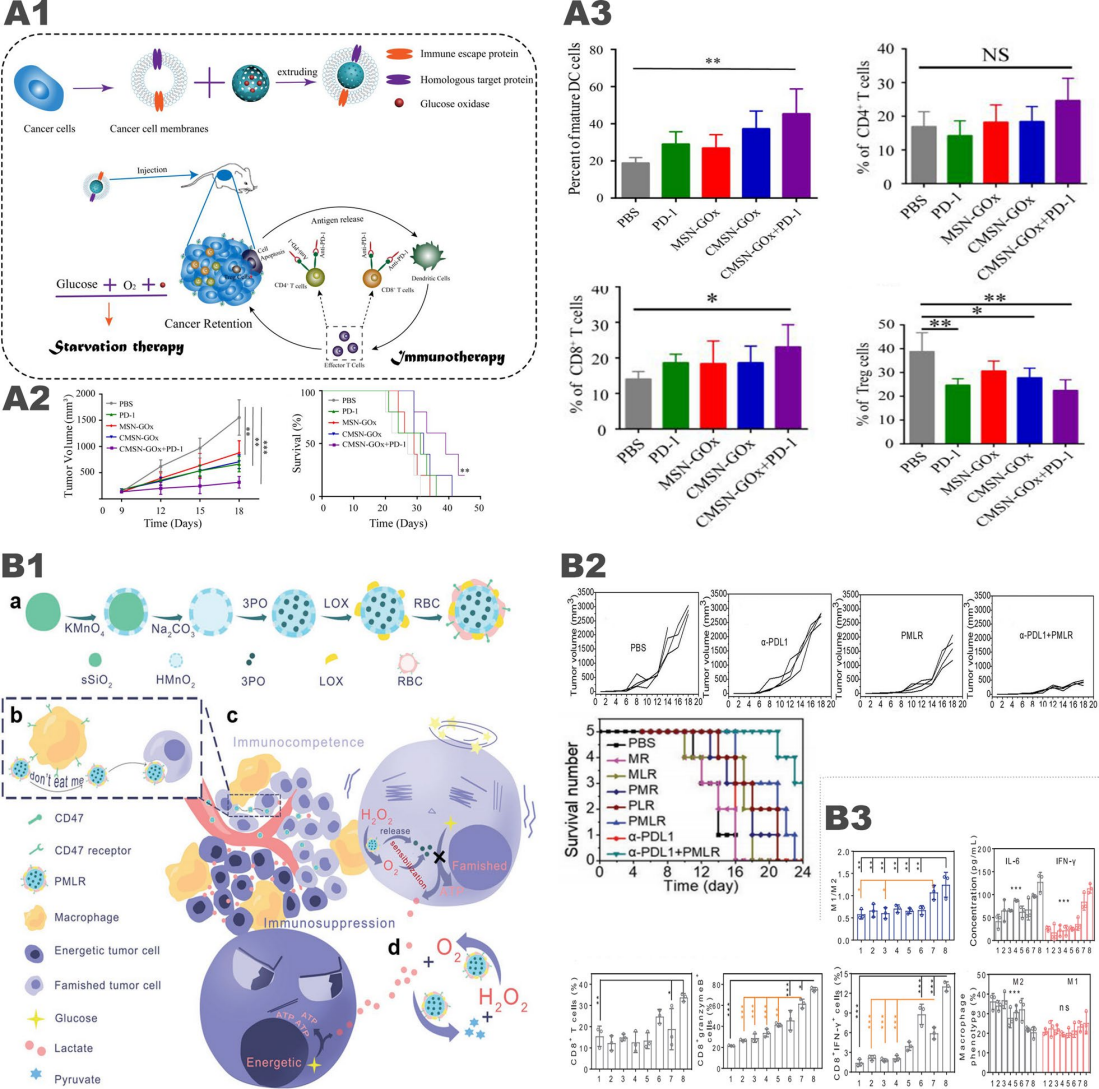
Oxidases can relieve the immunosuppression of TME in many ways. **A** Glucose oxidase mediated immune activation. **A1** Schematic illustration of anti-tumor immune response and enhanced anti-PD-1 immunotherapy induced by CMSN-GOx. **A2** Tumor volume and survival curves in the mice after different treatments. **A3** CMSN-GOx nanoparticles can promote immune activation. Reproduced with permission from Ref [56], copyright © 2019 American Chemical Society. **B** Lactate oxidase mediated immune activation. **B1** Schematic illustration of the intra/extracellular lactic acid exhaustion process of PMLR nanosystem. **B2** Tumor growth curves and survival curves of the mice with different treatments. **B3** PMLR nanosystem induces tumor immunity by regulating the activity and number of various immune cells. Reproduced with permission from Ref [18], copyright © 2019 WILEY–VCH

Zou et al. [122] constructed artificial NK cells (aNKs) using red blood cell membrane recapping of perfluorohexane (PFC) and GOx for specific tumor killing and repolarization of M2 to M1 macrophages. aNKs directly kill tumor cells and release TAAs by consuming glucose and producing H_2_O_2_. The generated H_2_O_2_ can reeducate renegade macrophages to phagocytose tumor fragments, presenting TAAs to T cells and activating the immune system to inhibit tumor cells. In vitro and in vivo experiments have demonstrated that aNKs have potent effects on tumor suppression and immunoactivation. Similarly, Xie et al. [56] constructed CMSN-GOx by loading GOx onto mesoporous silica nanoparticles (MSNs) to compound tumor cell membranes (Fig. 13, A1). Experiments have shown that CMSN-GOx can ablate tumors and induce DCs maturation, further activating tumor immunity. CMSN-GOx combined with anti-PD-1 performed better antitumor effects compared with applying CMSN-GOx or anti-PD-1 alone (Fig. 13, A2–3).

Wang et al. [47] designed a self-degradable microneedle (MN) patch for adjuvant immunotherapy for cutaneum carcinoma. The patch can penetrate the epidermis painlessly and drown in the interstitial liquid, sustainably releasing anti-PD-1 and GOx in the acidic environment to effectively deliver its payload to the TME. In vivo, it has been proved that the single use of the MN patch inhibited tumor growth better than the same dose of intertumoral injection of anti-PD-1in melanoma mouse mode.

##### Lactate oxidase and immunotherapy

Excessive lactic acid in the TME as well as the lactate-induced acidic environment can resist tumor immunity by negatively regulating innate and adaptive tumor-infiltrating immune cells. Firstly, lactate blocks the differentiation of monocytes into DCs, further reducing their antigen presentation ability [61]. Secondly, lactate inhibits the antitumor activities of immune effector cells, including NKs [123, 124] and CTLs [62]. Finally, lactate promotes the infiltrations of immunosuppressive cells, including M2 macrophages-like tumor-associated macrophages [63], N2 neutrophils-like tumor-associated neutrophils [125], bone marrow-derived myeloid suppressor cells (MDSCs) [126] and Tregs cells [127], which effectively inhibits the ant-tumor immune responses and promote immune escape. Therefore, reducing the accumulation and acidification of lactate in the TME is an important link to activate tumor immunity [128].

Gao et al. [18] adopted red blood cell membrane (mRBC) wrapping with hollow manganese dioxide (HMnO2) nanoparticles as a carrier, loading with glycolytic inhibitors and LOX to form a cascade catalytic nanosystem (PMLR) (Fig. 13, B1). PMLR has been observed to have pivotal effects on lactate depletion both inside and outside of cells, as well as synergistic metabolic therapy and immunotherapy. Thanks to the long-cycling properties of mRBC, the PMLR can gradually accumulate at the tumor site via the EPR effect. Extracellularly, the nanosystem consumes lactate in the TME by LOX-catalyzed oxidation reaction. Intracellularly, the nanosystem releases glycolytic inhibitors to cut off the lactate source, and achieves antitumor metabolic therapy by blocking ATP supply. O_2_ produced by the PMLR nanosystem leads to sensitization both in extracellular and intracellular processes. PMLR can continuously remove lactate in solid tumors and remold an immunoactive TME both in vitro and in vivo. Furthermore, this TME regulatory strategy can effectively improve the antitumor effects of anti-PD-L1 treatments without need of immunoagonists to avoid autoimmunity (Fig. 13, B2–3).

#### Synergistic effects of “metal-oxidase” system and immunotherapy

Tumor metastasis is the major cause of tumor lethality. Chemokinetic therapy, PTT and starving therapy all mainly target on primary tumors while having unsatisfactory efficacy in metastatic tumors. Recent evidence suggests that tumor immunotherapy can inhibit primary tumors and correlated metastasis [113]. However, the low rate of tumor immune response limits the application of immunotherapy. The chapters above have shown that transition metals, GOx and LOX can activate tumor immunity in different ways. Therefore, we naturally came up with the idea of using the “metal-oxidase” cascade catalytic system with maximum effect to reverse immunosuppression in tumor tissues. It was shown that the “metal-oxidase” system can not only activate tumor cell iron death or apoptosis through the synergy of CDT and starving therapy, but also trigger immunogenic cell death (ICD). The TAAs released from dying tumor cells promote DCs maturation, cytokine secretions, effectively activating T cell-mediated immunotherapy and greatly strengthening the role of immunotherapy in inhibiting metastasis [111, 129, 130]. Moreover, the “metal-oxidase” system can also consume large amounts of glucose or lactate in the TME to reverse the immunosuppressive state [18, 131].

**Fig. 14:**
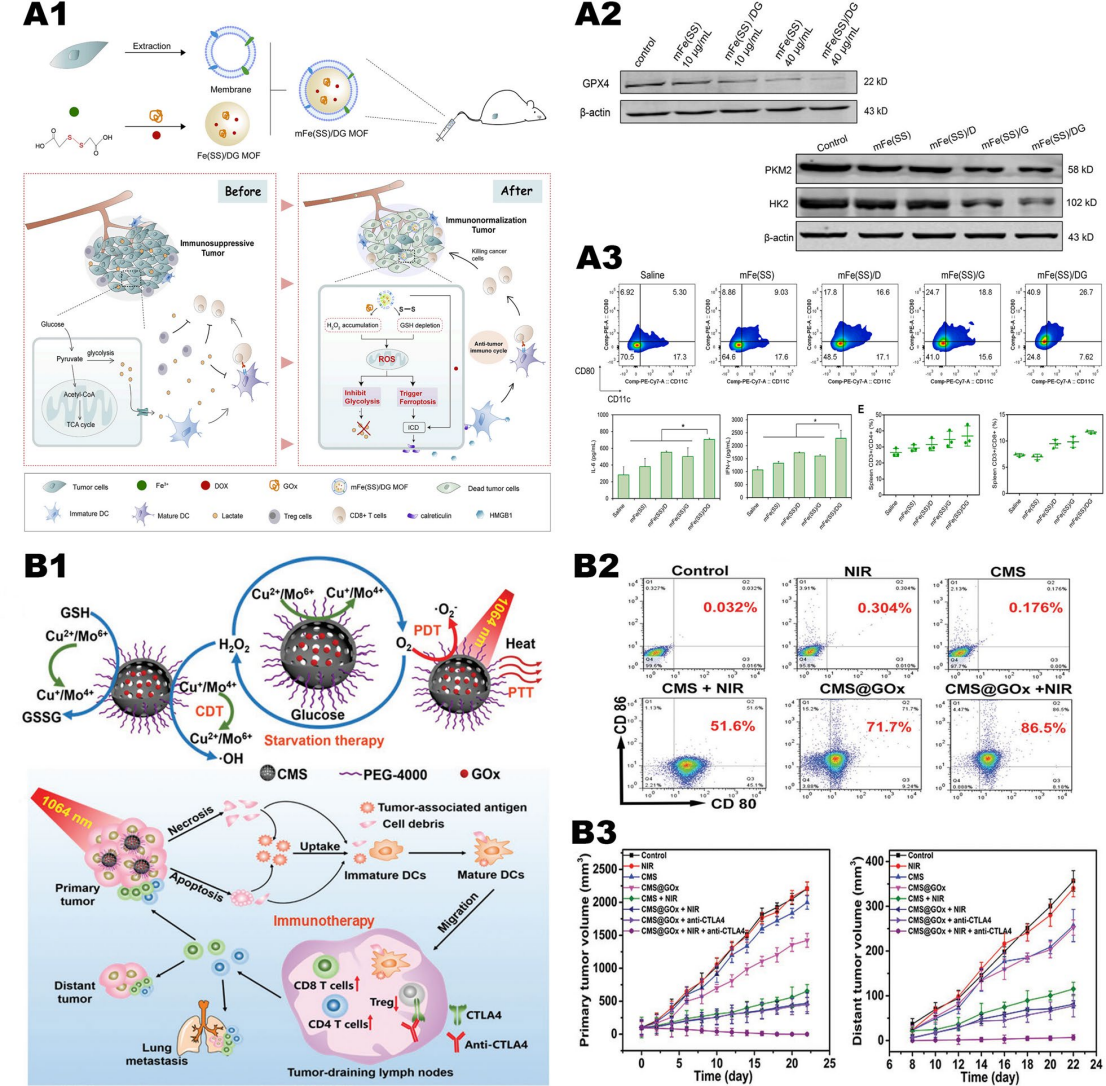
Synergistic effect of “metal-oxidase” cascade catalytic system and immunotherapy. **A1** Schematic illustration of the smart biomimetic nanoplatform for antitumor “metal-oxidase” and immunometabolism normalization based on the ROS-ferroptosis-glycolysis regulation. **A2** Measurement of GPX4, PKM2 and HK2 expression by western blot. **A3** ELISA analysis of IL-6 and IFN-γ levels and the relative quantification results of T cells of 4T1 tumor bearing mice after various treatments. The Reproduced with permission from Ref [19], copyright © 2021, Elsevier. **B1** Schematic illustration of fabrication and mechanism of PEGylated CMS@GOx for combined therapy. **B2** Quantification of CD80 and CD86 expression on the surface of human bone-marrow-derived DCs after different treatment by flow cytometry. **B3** Growth curves of primary tumor volume and distant tumor volume in Balb/c mice with different treatments. Reproduced with permission from Ref [136], copyright © 2019 WILEY–VCH

Ferroptosis is a recently discovered non-classical programmed cell death caused by the accumulation of lipid peroxidation products (LPO), leading in impaired cellular structure and integrity [132]. Yang et al. [19] synthesized a cancer cell membrane-coated metal organic frame (mFe (SS) /DG) loaded with GOx and DOX (Fig. 14, A1). The surface modification of MOF by the cancer cells membrane reduced MOF clearance and achieved homologous targeting of the tumors. The “metal-oxidase” system can effectively clear GSH and induce ROS bursts to downregulate glutathione peroxide 4 (GPX4), resulting in ferroptosis (Fig. 14, A2). Starvation effect, Ferroptosis combined with DOX induces tumor cell death and release of TAAs, which initiates antitumor immunity, subsequently undergoing the process of DCs maturation and presentation, further stimulating the CTLs proliferation. In vivo experiments showed that mature DCs levels were increased in the remaining groups compared to saline group (5.0%), with the highest one in mFe (SS)/DG group (26.7%). Moreover, IL-6 and IFN-γ secreted by tumor tissues were significantly increased in the mFe (SS)/DG group, which promoted immunity. Additionally, mFe (SS)/DG increased the number of helper CD4^+^ and cytotoxic CD8^+^ T cells compared with other groups (Fig. 14, A3). It is also pivotal that mFe (SS)/DG can avoid the lactate accumulation and reverse the acidic environment, thus reversing the immunosuppressive TME.

Apoptosis induced by the “metal-oxidase” system activates tumor immunity similarly. Shao et al. [20] synthesized IONP-GOD@ART for collaborative therapy using GOx-modified mesoporous iron oxide nanoparticles (IONP) loaded with artemisinin (ART). In an acidic environment, the nanomaterials gradually decompose and release Fe^2+^/Fe^3+^, GOx, and ART. GOx form a “metal-oxidase” cascade catalytic system with Fe^2+^. In addition, unstable endoperoxide bridge is disrupted in ART in the presence of Fe^2+^, producing a large number of reactive oxygen species (ROS). The generated ROS further triggers tumor cell death and the release of TAAs to activate tumor immunity. Experiments showed that IONP-GOD@ART can induce about 40% of DCs to mature, far beyond other control groups. It can also induce repolarization of M2 macrophages to M1 macrophages, while observing an increase of IL-12 (produced by M1 macrophages) and decrease of IL-10 (produced by M2 macrophages). In vivo experiments have shown that IONP-GOD@ART can completely inhibit tumor growth and distant metastasis, ensuring biosafety while having immune activation effect.

Although the “metal-oxidase” system above can kill tumor cells and release TAAs, further inducing DCs maturation. However, the overexpressed prostaglandin E2 (PGE2) in tumors prevented the migration of DCs and further inhibited CTLs activation and tumor infiltration [133]. Aspirin (ASA) was found to improve the immune microenvironment [134] by reducing the secretion of PGE2 via inhibiting COX-2 expression. Sun et al. [135]  constructed the Au@HMnMSNs nanoplatform loading DOX and ASA. Nanoparticles can consume GSH in an acidic environment and release Mn^2+^ to further induce Fenton-like reactions, while the inner gold nanoparticles can convert glucose into H_2_O_2_ to accelerate •OH generation with inducing a starving effect. ASA was introduced to aggregate DCs and CTLs into tumor tissues and promote tumor immunity by reducing PGE2 expression. In vitro assays, CRT and HMGB1 release significantly increased in 4T1 cells after Au@HMnMSNs treatment, suggesting that the cascade of catalysis has induced tumor cell death and released TAAs. Simultaneously, DCs maturation increased significantly after nanoparticle treatment by up to 57.1% as detected by flow cytometry. Alaltered secretion of numerous cytokines was also observed, in which significantly increased cytokines were immuno-promoted (IL-6, IL-12p70, IFN-γ and TNF-α). In contrast, the levels of immunosuppressive cytokines (IL-10) are reduced. In addition, ASA acted as expected, with significantly downregulated expression levels of COX-2 and PGE2 in 4T1 cells, which also promoted DCs maturation. In in vivo experiments, the infiltration abundance of helper CD4^+^ and cytotoxic CD8^+^ T cells was significantly increased in mice tumor tissues treated with nanoparticles. The combination of the “metal-oxidase” system with ASA can not only completely eliminate primary tumors but also activate tumor immunity to inhibit tumor metastasis.

The above studies show that the “metal-oxidase” can act as immune agonists. So we naturally think of using immune checkpoint inhibitors in combination with the “metal-oxidase” system, in which the former is responsible for relieving the immunosuppression in the TME while the latter is responsible for further activating tumor immunity. Only the two cooperate with each other can they achieve the ideal treatment effects. A multifunctional cascade bioreactor (CMS@GOx) based on hollow mespore Cu_2_MoS_4_ (CMS) loading GOx was constructed by Chang et al. [136] (Fig. 14, B1). CMS contains multivalent transition metal elements (Cu^+^/^2+^, Mo^4+^/^6+^) with both glutathione peroxidase activity and catalase activity. Cu^2+^/Mo^6+^ can reduce the antioxidant capacity of tumors by consuming overexpressed GSH in the TME, simultaneously producing Cu^+^ and Mo^4+^ to catalyze the Fenton-like reaction. CMS can also react with H_2_O_2_ to generate O_2_, activating GOx catalytically oxidized glucose. In addition, CMS has a good photothermal conversion efficiency (η = 63.3%) and generates cytotoxic superoxide anions (•O_2_^−^), at 1064 nm of near-infrared light irradiation. In vitro experiments, CMS@GOx in combination with 1064 nm laser irradiation significantly induced DCs maturation (86.5%) and induce DCs to release a variety of immune-promoting cytokines. In in vivo experiments, when further combined with CTLA-4, the primary and metastasis sites of the tumor were completely inhibited while substantial helper CD4^+^ and cytotoxic CD8^+^ T cells infiltrations were detected in the metastatic sites. In addition, Chang et al. also injected U14 cancer cells IV into mice treated with both CMS@GOx and CTLA-4 and mice untreated. The results showed that the untreated mice developed substantial lung metastases, while the treated mice with permanent immunity had almost no pulmonary nodules. This indicates that the “metal-oxidase” cascade catalytic system will produce extremely powerful permanent tumor immunity in combination with immune checkpoint inhibitors, which can not only completely eliminate the tumor primary sites and metastasis but also prevent tumor recurrence.

## Current status of nanomaterials for drug delivery

The EPR effect can be regarded as a landmark discovery in nanomedicine. The imbalance between nutritional supply and demand in mature solid tumors leads to the formation of massive abnormal neovascularization. The large spacing of endothelial cells in this neovascularization is conducive to the magnanimous aggregation of nanomaterials in tumor tissues, which is exactly the EPR effect [137] introduced above. Targeted drug delivery based on the EPR effect is called passive targeted drug delivery. The size, shape and surface charge of the nanoparticles are closely correlated with the efficiency of the drug delivery system [138, 139], in which the nanoparticles with a diameter of 20–200 nm are most likely to achieve the EPR effect [140].

However, the effectiveness of the EPR effect has become controversial in recent years. The researchers found that there was no supply and demand imbalance in the initial solid tumors and thus absent of a large number of abnormal blood vessels, resulting in the failure of the EPR effect [141]. Sindhwani et al. found that only a few nanomaterials enter tumors through vascular endothelium while up to 97% of nanoparticles enter tumours via an active process through endothelial cells [142]. Cheng et al. [143] recently analyzed the published nanomedicine literature (2005–2018) and revealed that only 0.76% of the systemically applied nanoparticles successfully reached solid tumors on average. Despite a large number of nanomaterials have been proved to perform excellent antitumor effects in mouse models, only 10 of them were approved for clinical applications in tumor treatment. In addition, only 14% of the nanomaterials reaching the phase III clinical trials have successfully proven efficacy [144].

Even if the EPR effect indeed exists, there are few nanoparticles successfully making it to the tumor site. In addition to relying on the EPR effect present in mature tumors, the researchers found that nanomaterials could also enhance their enrichment in tumor tissues via inducing vascular endothelial leakage [145]. Setyawati et al. [146] identified another form of endothelial leakage unrelated to cytotoxic and oxidative stress and described this phenomenon as nanomaterial induced endothelial leakiness (NanoEL). This form of endothelial leakage mainly depends on vascular cadherin (VE-cadherin) destructions, coupled with actin remodeling and cell contractions to expand the intercellular gap by at least 1 mm. By far, numerous studies have shown that some inorganic nanomaterials can form micron-sized gaps between vascular endothelial cells via NanoEL effect under the circumstance of certain optimal size, surface charge and density. Peng et al. [147] found that intravenous injection of TiO_2_ NPs, Au NPs and SiO_2_ NPs promoted vascular endothelial leakage in breast cancer. Setyawati et al. [148] found that the smaller the size of Au NPs were, the higher the inductivity of endothelial leakage showed. The surface charge of Nanoparticles also interferes with the NanoEL effect. By Synthetizing Au NPs with different surface charges, Wang et al. intriguingly observed that [149] the negatively charged Au NPs cause a greater NanoEL effect than the positively charged Au NPs. Furthermore, Tay et al. [150] observed that the NanoEL effect raised by SiO_2_ NPs depends on the density of NPs to a certain extent. Unlike the EPR effect relying on abnormal angiogenesis in mature solid tumors, the NanoEL effect can be induced by the innate capacities of nanomaterials. This means that well-designed nanomaterials can actively induce the leakage of vascular endothelial cells to pass through the blood vessels and aggregate substantially in the tumor tissues, regardless of tumor type and stage.

Almost all of the “metal-oxidase” systems rely on the EPR effect to achieve passive drug delivery, which is undoubtedly an inefficient way. At present, the mature nanoparticles used for inducing the NanoEL effect mainly include Au NPs, SiO_2_ NPs and TiO_2_ NPs. We made a consumption based on the existing literature: with reasonable design, whether it is possible to adopt SiO_2_ as nanovector or add Au NPs in the system to synthesize the “metal-oxidase” nanoplatform with NanoEL effect. The “metal-oxidase” nanoplatform with NanoEL effect can excessively accumulate in tumor tissues, further acting to eliminate tumor cells. However, the induction of vascular endothelial leakage by nanomaterials through NanoEL effect may lead to a subset of side effects, most important ones among which are the promotion of blood metastasis of tumors [146, 147], the aggravation of bacterial infections [151] and the boost of edema and thrombosis [152]. Thus, we are supposed to take these factors into account in the design of “metal-oxidase” system with NanoEL effect to minimize their adverse effects [153].

## Summary and outlook

At present, the use of transition metal ions to catalyze Fenton/Fenton-like reaction to induce CDT is a gradually mature and promising tumor treatment strategy. Iron, copper and manganese are common catalyzing Fenton/Fenton-like metals. As Fe^2+^/Fe^3+^ has strong catalytic ability and high biological safety, iron-based nanoparticles are considered as the best option. However, the optimal pH value of Fe^2+^/Fe^3+^ is low (pH 2 ~ 4), which is difficult to achieve in untreated TME. Compared with Fe^2+^/Fe^3+^, Cu^+^/Cu^2+^ has a higher catalytic efficiency and a wider pH range. At the same time, Cu^+^/Cu^2+^ can remove GSH to enhance the killing effect of ROS, but the accumulation of copper ions will cause greater damage to human body, thus more attention needs to be paid to biological safety. Mn^2+^ shows effective catalytic ability in the entire pH range. In addition, Mn^2+^ can effectively promote immunity and is a severely “ignored” nanomaterial. Transition metal can catalyze the Fenton/Fenton-like reaction and generate strong oxidizing •OH to further kill tumor cells. Sufficient substrate and appropriate pH are the basis of the Fenton/Fenton-like reaction. However, the lack of endogenous H_2_O_2_ in tumor tissues and insufficient acidity of the TME are undoubtedly the most critical factors limiting CDT. Therefore, we need to find a novel tumor treatment strategy which provides strong complementarities to CDT, exerting a stronger synergistic effect by combining with CDT.

Biological metabolite oxidases (e.g., GOx, LOX, AAO) can consume the nutrients and acidify the TME with generation of excessive H_2_O_2_, inducing starvation therapy for tumor treatment. For instance, GOx can oxidative and decompose glucose and inhibit glycolysis, inhibiting the energy supply of tumor cells from two perspectives, further inducing starvation therapy. Meanwhile, acidified TME can be used to activate pH sensitive drug delivery vector to continuously release antitumor drugs. Most importantly, a large amount of strongly oxidative H_2_O_2_ is generated, which obviously increases intratumor oxidative stress and then leads to cell death. Studies have shown that lactate is also one of the energy sources of tumor cells, promoting tumor proliferation and inhibiting tumor immunity through multiple pathways. Therefore, the use of LOX can reverse the suppressive tumor immune microenvironment while inducing tumor starvation therapy and oxidative therapy. Similarly, amino acids are fundamental for protein synthesis, as well as an important energy source for tumor cells. AAO also induces tumor starvation therapy and breaks metabolic balance within tumor cells. In most solid tumors, the oxygen consumption of oxidases largely aggravates the original hypoxia in the TME. However, adequate oxygen is necessary for the functions of intracellular metabolite oxidases. Therefore, combining oxidases with carriers or drugs which promote oxygen generation can achieve ideal antitumor effects.

Treating with CDT or starvation therapy alone has lots of limitations, and their antitumor efficacy is not satisfactory as well. Naturally, we associate CDT with starvation therapy to produce a “metal-oxidase” cascade catalytic system (such as Fe- GOx, Cu-GOx, Fe-LOX, and Fe-AAO). On the one hand, GOx induces starvation therapy and generates a large amount of endogenous H_2_O_2_, which can be used as the substrate of CDT to further generate more oxidizing •OH, and acidify the TME to form a more suitable condition for Fenton/Fenton-like reaction. On the other hand, natural GOx has defects including poor stability, short half-life and immunogenicity. And intravenous administration of GOx will lead to the systemic production of H_2_O_2_, resulting in significant adverse effects. Therefore, designing appropriate metal-based nanovectors to protect GOx can prolong its circulation time and greatly improve its biosafety. Therefore, the “metal-oxidase” system can combine their advantages to form a mutual-promoted cascade catalytic system. At present, this cascade catalytic system has potential in tumor treatment, but we still need to modify it to realize its maximum effect. For example: (1) By adding reducing substances such as TA into the system, Fe^3+^ can be reduced into Fe^2+^ with stronger catalytic ability, so as to enhance Fenton reaction. (2) By adding certain nanoparticles to induce PTT can facilitate the cascade catalytic system. (3) By adopting nanovectors with peroxidase activity (such as MnO_2_) can react with H_2_O_2_ in the TME to generate oxygen, sensitizing GOx and increasing glucose consumption to further improve the efficacy of the cascade catalytic system.

Based on the “metal-oxidase” cascade catalytic system above, people have developed a “metal-oxidase-chemotherapeutic drug” comprehensive treatment system. At present, the added chemotherapeutic drugs include CPT, TPZ, DOX, etc. Compared with the direct use of chemotherapeutic drugs, the use of “metal-oxidase” nanovectors to deliver chemotherapeutic drugs has the following advantages: (1) The targeted enrichment in tumors can enhance the specific killing of tumors. (2) Chemotherapy drugs are protected by nanovectors before reaching the tumor tissue, which greatly reduces the significant adverse effects. (3) Due to the anti-tumor effects of the nanoparticles, smaller doses of chemotherapeutic drugs could lead to better tumor ablation. (4) It is possible to exert better efficacy because of the prolonged circulation time of the chemotherapeutic agents. (5) Nanomaterials can remold the TME, overcoming drug resistances of chemotherapeutic agents. Based on the existing studies, we found that the “metal-oxidase-chemotherapeutic drug” comprehensive treatment system has valid antitumor effect and reliable biosafety. The hypoxia of TME will be aggravated in the process of starvation therapy induced by oxidases, which is one of the limitations of the application of oxidases. However, hypoxia-activating drugs only act in hypoxic environment. Therefore, the combined use of hypoxia-activating drugs with the “metal-oxidase” system makes hypoxia an advantage, maximizing the effects of hypoxia-activating drugs and improves their biosafety. In addition, most metal-based nanoparticles have good photothermal effect, so “metal-oxidase” combined PTT has natural advantages. On the one hand, the mass heat can directly induce tumor cell death. On the other hand, it greatly accelerates the cascade catalytic reaction.

More and more researchers focus on tumor immunotherapy and believe that immunotherapy is an effective strategy to inhibit tumor metastasis and recurrence. Current studies have shown that both metal-based nanoparticles and oxidases can further promote DCs maturation and activate tumor immunity by inducing tumor cell death and releases of TAAs. At the same time, GOx and LOX can remold the inhibitory immune microenvironment (such as reducing lactate accumulation). The treatment of “metal-oxidase” system combined with immunotherapy derived on the basis of these findings. The mechanisms of the synergistic effect are as follows: (1) Metal-based nanoparticles such as Mn^2+^ can independently activate immunity. (2) GOx and LOX can avoid lactate accumulation and increase pH, reversing the immune suppression in the TME. (3) The “metal-oxidase” cascade catalytic system can strongly eliminate tumor cells and release TAAs to enhance the antigen presentation and the recruitment of antigen-presenting cells, promoting the infiltration of CTLs in tumor tissues. Tumor metastasis is the main cause of tumor death. Studies have shown that “metal-oxidase” system combined with immunotherapy can completely eliminate the primary site of tumor, effectively inhibiting tumor metastasis and recurrence. Therefore, the “metal-oxidase” system will become the next hot spot in the future.

Almost all of the “metal-oxidase” systems rely on EPR effect to achieve passive drug delivery, which is undoubtedly an inefficient way. Different from EPR effect, nanoparticles can actively induce vascular endothelial leakage and further generate millimeter-level gaps independently on the basis of NanoEL effect, no longer relying on abnormal vessels formed in mature solid tumors. However, NanoEL effect may also lead to a series of side effects, the most important one among which is to promote tumor blood metastasis. Therefore, while designing the “metal-oxidase” nanosystem with NanoEL effect, we should consider the problems above to minimize its adverse effects.

In conclusion, both CDT based on metal catalysis and starvation therapy based on oxidase catalysis can be used in tumor treatment, but they have limitations. Metal-nanovectors have acquired valid synergistic effect after loading oxidases, forming a “metal-oxidase” cascade catalytic system and showing powerful antitumor effects and reliable biosafety. At the same time, “metal-oxidase” system can cooperate with chemotherapy, hypoxia-activated drugs, PTT, and immunotherapy to maximize the value of the system.

## Data Availability

Not applicable.
